# Efficacy and Safety of Ginkgo Diterpene Lactone Meglumine in Acute Ischemic Stroke: A Randomized Clinical Trial

**DOI:** 10.1001/jamanetworkopen.2023.28828

**Published:** 2023-08-14

**Authors:** Qian Zhang, Anxin Wang, Qin Xu, Xue Xia, Xue Tian, Yijun Zhang, Xiaolong Li, Xiusheng Yang, Xingchen Wang, Jinghua Peng, Yanchun Li, Luran Liu, Shunshan Jin, Xia Meng, Xingquan Zhao

**Affiliations:** 1 Department of Neurology, Beijing Tiantan Hospital, Capital Medical University, Beijing, China; 2 China National Clinical Research Center for Neurological Diseases, Beijing Tiantan Hospital, Capital Medical University, Beijing, China; 3 Department of Epidemiology and Health Statistics, School of Public Health, Capital Medical University, Beijing, China; 4 Beijing Municipal Key Laboratory of Clinical Epidemiology, Beijing, China; 5 Department of Neurology, Xiangyang No. 1 People’s Hospital, Hubei, China; 6 Department of Neurology, The First People’s Hospital of Yuanping City, Shanxi, China; 7 Department of Neurology, The Second Affiliated Hospital of Shandong University of Traditional Chinese Medicine, Shandong, China; 8 Department of Neurology, Jiujiang No. 1 People’s Hospital, Jiangxi, China; 9 Department of Neurology, The First Affiliated Hospital Of Hunan University of Medicine, Hunan, China; 10 Department of Neurology, The Fourth Hospital of Harbin Medical University, Heilongjiang, China; 11 Department of Neurology, Beidahuang Group General Hospital, Heilongjiang, China; 12 Research Unit of Artificial Intelligence in Cerebrovascular Disease, Chinese Academy of Medical Sciences, Beijing, China; 13 Beijing Institute of Brain Disorders, Collaborative Innovation Center for Brain Disorders, Capital Medical University, Beijing, China

## Abstract

**Importance:**

Ginkgo diterpene lactone meglumine (GDLM) has attracted much attention because of its potential neuroprotective properties in ischemic stroke. The efficacy of GDLM in patients with acute ischemic stroke (AIS) needs to be verified by well-designed randomized clinical trials.

**Objective:**

To assess the efficacy and safety of GDLM in patients with AIS.

**Design, Setting, and Participants:**

This multicenter, randomized, double-blind, placebo-controlled, parallel-group trial involved 3448 patients who had AIS, were aged 18 to 80 years, had a clinically diagnosed AIS symptom within 48 hours of onset, had a modified Rankin Scale (mRS) score of 0 or 1 prior to onset, and had a National Institutes of Health Stroke Scale score ranging from 4 to 24. The trial took place at 100 centers in China from February 1, 2016, to May 1, 2018. The mRS is a global stroke disability scale with scores ranging from 0 (no symptoms or completely recovered) to 6 (death). The National Institutes of Health Stroke Scale is a tool used by clinicians to quantify impairment caused by stroke (range, 0-42, with higher scores indicating greater severity). Data were analyzed from January 2019 to December 2022.

**Interventions:**

Patients were randomized to receive GDLM or placebo once daily via intravenous infusion in a 1:1 ratio. The treatment was dispensed within 48 hours after symptoms and continued for 14 days. Interventions of thrombolysis and thrombectomy were not permitted during the treatment.

**Main Outcomes and Measures:**

The primary outcome was the proportion of patients with an mRS of 0 or 1 on day 90 after randomization. Safety outcomes included adverse events and serious adverse events.

**Results:**

A total of 3448 patients were randomized, with 1725 patients assigned to the GDLM group and 1723 patients assigned to the placebo group. The median (IQR) age of the patients was 63 (55-71) years, and 1232 (35.7%) were women. The primary outcome on day 90 occurred in 877 patients (50.8%) in the GDLM group, and 759 patients (44.1%) in the placebo group (risk difference, 6.79%; 95% CI, 3.46%-10.10%; odds ratio, 1.31; 95% CI, 1.15-1.50; relative risk, 1.15; 95% CI, 1.08-1.24; *P* < .001). Adverse events occurred relatively equally between the 2 groups (303 [17.6%] vs 298 [17.3%]; risk difference, 0.27%; 95% CI, −2.26% to 2.80%; odds ratio, 1.02; 95% CI, 0.85-1.21; relative risk, 1.02; 95% CI, 0.88-1.17; *P* = .83).

**Conclusions and Relevance:**

Among patients with AIS in this randomized clinical trial, GDLM improved the proportion of patients achieving favorable clinical outcomes at 90 days compared with placebo.

**Trial Registration:**

ClinicalTrials.gov Identifier: NCT02526225

## Key Points

**Question:**

Does ginkgo diterpene lactone meglumine (GDLM) improve the functional outcome at 90 days in patients with acute ischemic stroke (AIS)?

**Findings:**

This randomized clinical trial included 3448 patients with AIS who received GDLM or placebo injections within 48 hours after symptoms for 14 days; the proportion of patients achieving a favorable outcome, defined as the 90-day modified Rankin Scale score of 1 or 0, was 50.8% in the GDLM group, significantly higher than 44.1% in the placebo group. The rate of adverse events was similar between the 2 groups.

**Meaning:**

Among patients with AIS, ginkgo diterpene lactone meglumine improved the proportion of patients achieving favorable clinical outcomes at 90 days compared with placebo.

## Teaser

This randomized clinical trial assesses the safety and functional outcomes at 90 days of ginkgo diterpene lactone meglumine, a neuroprotective agent, in patients with acute ischemic stroke.

## Introduction

Neuroprotection has emerged as a potential therapeutic approach in patients with acute ischemic stroke (AIS). Multiple pathological and physiological processes are involved in the occurrence and progression of AIS, including excitotoxicity, oxidative and nitrosative stress, cell apoptosis, and inflammation.^1,2^ One or more of these molecular targets were selected for therapeutic intervention in a number of neuroprotectant trials.^3,4,5,6,7^ Ginkgo diterpene lactone meglumine (GDLM) is made of extracts from ginkgo biloba L, composed of the active ingredients of ginkgolides A, B, and K, and some other contents. It has been widely used in the treatment of ischemic stroke in China.^8,9,10^ The active ingredient in GDLM has been shown to have a variety of neuroprotective and reparative effects that can help maintain the blood-brain barrier; reduce brain edema; improve energy metabolism; help with antioxidation, anti-inflammation, and antiapoptosis; and promote angiogenesis.^9,11,12,13^ Several clinical studies have evaluated the efficacy of GDLM in the treatment of stroke,^11,14^ whereas most of these studies were limited by small sample size, single-center design, and no placebo-controlled group to compare the single therapeutic response with GDLM. Therefore, it is still of great importance to verify the therapeutic value of the multitargeted GDLM therapuetic in well-designed randomized clinical trials with a sufficient numer of patients with AIS. In this multicenter, double-blinded, placebo-controlled trial, we aimed to evaluate the efficacy and safety of GDLM in patients with AIS compared with placebo.

## Methods

### Trial Design

This was a multicenter, randomized, double-blind, placebo-controlled, parallel-group trial conducted at 100 centers in China from February 1, 2016, to May 1, 2018. Details of the trial rationale, design, and methods were provided in the trial protocol (Supplement 1). The trial design was approved by the ethics committee at Beijing Tiantan Hospital and each participating site. Written informed consent for participation in the trial was provided by the patients or their legal representative. The trial was conducted in accordance with the principles of the Declaration of Helsinki and followed the Consolidated Standards of Reporting Trials (CONSORT) reporting guideline. Data were analyzed from January 2019 to December 2022.

The steering committee was responsible for the design and supervision of the trial, the development of and amendments to the protocol, and the interpretation of the data. The steering committee was also responsible for ensuring the integrity of the data, analysis, presentation of results, and the fidelity of the trial to the protocol. An independent clinical event adjudication committee, whose members were unaware of the trial group assignments, adjudicated the primary and secondary efficacy outcomes and bleeding events. An independent data and safety monitoring committee monitored the progress of the trial, with regular assessment of safety outcomes, overall trial integrity, and trial conduct.

### Patient Eligibility Criteria

Patients were eligible if they were aged 18 to 80 years, had a clinically diagnosed AIS symptom within 48 hours of onset, had a modified Rankin Scale (mRS) score of 0 or 1 prior to onset, had a National Institutes of Health Stroke Scale (NIHSS) score between 4 and 24 after onset (indicating moderate stroke), had an upper and lower limb motor deficit score on the NIHSS of at least 2, and signed informed consent. The mRS is a global stroke disability scale with scores ranging from 0 (no symptoms or completely recovered) to 6 (death). The NIHSS is a tool used by clinicians to quantify impairment caused by stroke (range, 0-42, with higher scores indicating greater severity). The motor arm and leg deficits are subcategories of the NIHSS, scored on a 0 to 4 scale, with higher scores indicating greater severity. The detailed exclusion criteria are shown in eTable 1 in Supplement 2.

### Randomization and Blinding

Within 48 hours after symptom onset, eligible patients were randomly assigned in a 1:1 ratio to receive GDLM or placebo by a computerized block randomization method with randomly selected block sizes of 4. The randomization number was stimulated centrally by an independent statistician. The 2 forms of drugs were visually identical and cannot be distinguishable in appearance. Both researchers and patients were blinded to the treatment.

### Treatment

Patients in the GDLM group received a GDLM injection of 5 mL (active ingredient GDLM, 25 mg) once daily via intravenous infusion for 14 consecutive days. Patients in the placebo group received a GDLM mimic injection (physiological saline) of 5 mL once daily via intravenous infusion for 14 consecutive days. Both the GDLM and placebo were diluted in 250 mL of sterile 0.9% sodium chloride injection. Treatment was dispensed within 48 hours after symptoms. Treatment was discontinued when the patients were discharged prior to 14 days. All of the patients were followed up to day 90 after randomization.

### Outcomes

The primary efficacy outcome was the proportion of patients with a mRS score of 0 or 1 on day 90 after randomization. The secondary outcomes included the proportion of patients with an mRS score of 2 or less on day 90 after randomization, the proportion of patients with a decrease in NIHSS score of at least 4 points from baseline to day 7 and day 14 after randomization, the proportion of patients with an increase in NIHSS score of 4 or more points, 3 points, 2 points, or 1 point from baseline to day 7 after randomization. Safety outcomes, including adverse events, serious adverse events within 90 days, changes in vital signs, and results of laboratory examinations (routine blood, routine urine, and blood biochemistry examinations) from baseline to day 14 after randomization were systematically recorded.

### Statistical Analysis

We determined that a total of 3452 patients would provide 80% power to detect a 45% rate of patients with mRS scores of 0 or 1 on day 90 after randomization in the placebo group^15^ and a 50% rate in the GDLM group with a 2-sided significance level of *P* < .05 and an overall dropout rate of approximately 10%.

All the analyses were performed in the modified intention-to-treat population, which comprised all the patients who had undergone randomization and had at least 1 assessment of efficacy after baseline. Baseline data were presented according to treatment assignment. Continuous variables were presented as mean (SD) or median (IQR) according to the distribution, and categorical variables were presented as frequency and proportion. The differences in the proportions for the dichotomous outcomes between treatment groups and their corresponding 95% CIs were estimated based on the Newcombe-Wilson score method.^16^ Missing data on the primary outcome were dealt with by using the last observation carried forward method. The primary and secondary outcomes were assessed with logistic regression and odds ratios (ORs), and 95% CIs were reported. Relative risk (RR) was assessed using a generalized linear model with a logarithmic link. The same methods were used in the per-protocol analysis. In addition, the treatment effects (primary outcome) were analyzed among several prespecified subgroups (age, sex, previous stroke, hypertension, diabetes, and time from onset to treatment). Missing data on the primary outcome were also assessed with the multiple imputation technique in the sensitivity analysis. All statistical tests were performed using 2-sided tests, and *P* < .05 was considered statistically significant. Statistical analyses were performed with SAS statistical software, version 9.4 (SAS Institute).

## Results

### Baseline Characteristics

**Figure 1.**
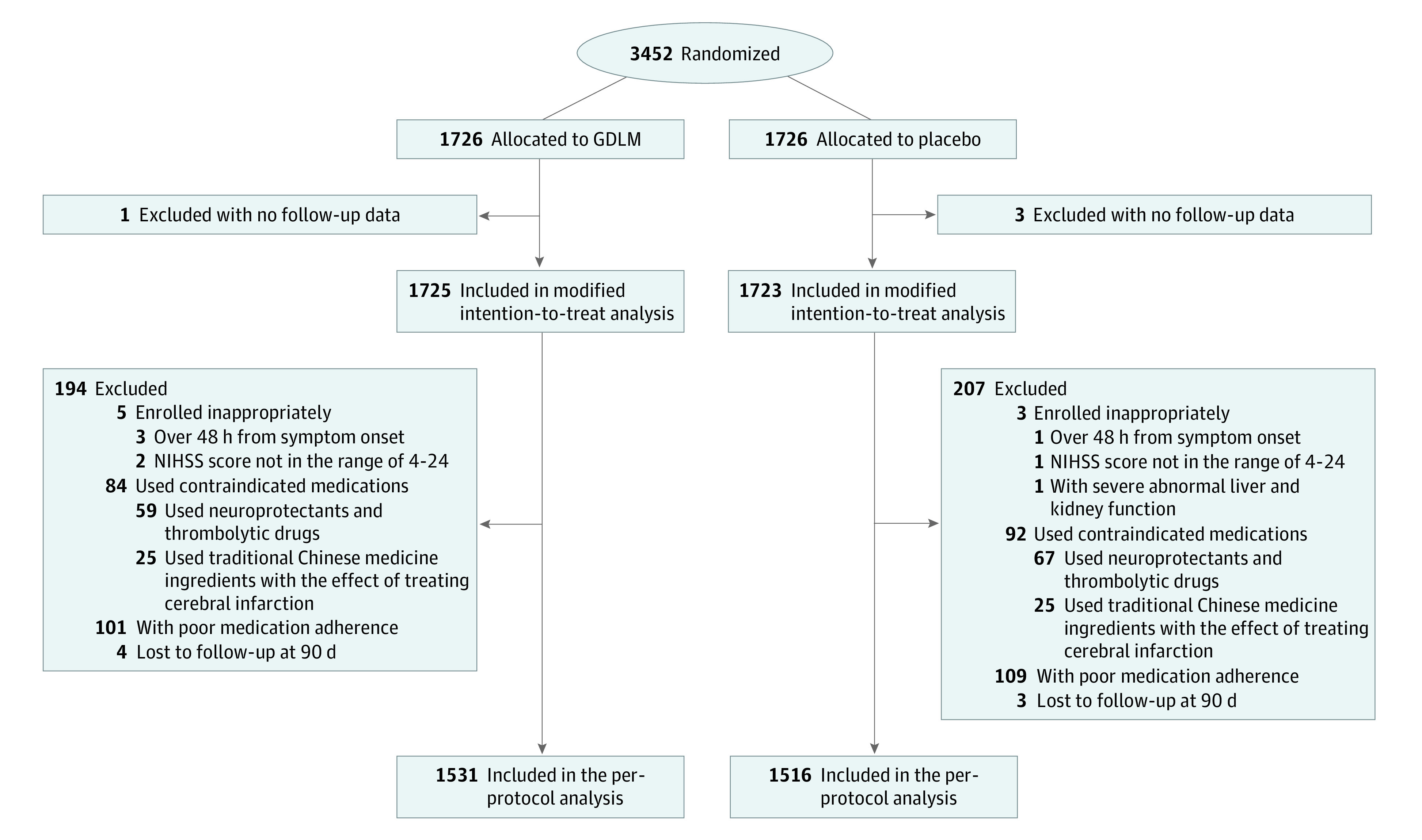
Flowchart of the Study Abbreviations: GDLM, ginkgo diterpene lactone meglumine; NIHSS, National Institutes of Health Stroke Scale. The NIHSS is a tool used by clinicians to quantify impairment caused by stroke (range, 0-42, with higher scores indicating greater severity).

A total of 3452 patients with AIS were enrolled at 100 centers in China. A total of 1726 were assigned to receive GDLM, and 1726 were assigned to receive placebo (Figure 1). One participant in the GDLM group and 3 in the placebo group (without any follow-up data) were excluded, leaving 3448 patients in the modified intention-to-treat analysis. Then 8 participants were excluded who enrolled inappropriately, 149 were excluded with contraindicated medications, 209 were excluded with poor medication adherence (compliance outside the range of 80%-120%), and 7 were excluded because they were lost to follow-up at 90 days with missing data on the primary outcome, leaving 3075 patients in the per-protocol analysis.

The characteristics of the patients at baseline were well balanced between the 2 groups (Table 1). The median (IQR) age of the patients was 63 (55-71) years. A total of 1232 (35.7%) were women. The median (IQR) baseline NIHSS score was 7 (6-9) points across all participants. The median (IQR) time from stroke onset to treatment was 24 (10-31) hours. Concomitant treatment and prohibited medications taken within 90 days are presented in eTable 2 and eTable 3 in Supplement 2.

**Table 1.** Baseline Characteristics

Characteristic	No. (%)	
	GDLM group (n = 1725)	Placebo group (n = 1723)
Age, median (IQR), y	63 (55-71)	63 (54-70)
<65 y	930 (53.9)	944 (54.8)
≥65 y	795 (46.1)	779 (45.2)
Sex		
Female	640 (37.1)	592 (34.4)
Male	1085 (62.9)	1131 (65.6)
Body mass index, median (IQR)^a^	23.7 (21.8-25.6)	23.8 (21.9-25.7)
Systolic blood pressure, median (IQR), mm Hg	146 (132-160)	144 (130-160)
Diastolic blood pressure, median (IQR), mm Hg	86 (80-96)	86 (80-94)
Medical history		
Previous stroke	394 (22.8)	380 (22.1)
Hypertension	1058 (61.3)	1055 (61.2)
Diabetes	399 (23.1)	366 (21.2)
Time from onset of stroke to treatment, median (IQR), h	24 (10-31)	24 (10-31)
Time to randomization, h		
<24	740 (42.9)	743 (43.1)
≥24	985 (57.1)	980 (56.9)
NIHSS score, median (IQR)^b^	7 (6-9)	8 (6-9)
NIHSS score categories^b^		
≤7	892 (51.7)	859 (49.9)
>7	833 (48.3)	864 (50.1)

Abbreviations: GDLM, ginkgo diterpene lactone meglumine; NIHSS, National Institutes of Health Stroke Scale.

^a^ Body mass index is calculated as weight in kilograms divided by height in meters squared.

^b^ The NIHSS is a tool used by clinicians to quantify impairment caused by stroke (range, 0-42, with higher scores indicating greater severity).

### Outcomes

#### Primary Outcome

**Figure 2.**
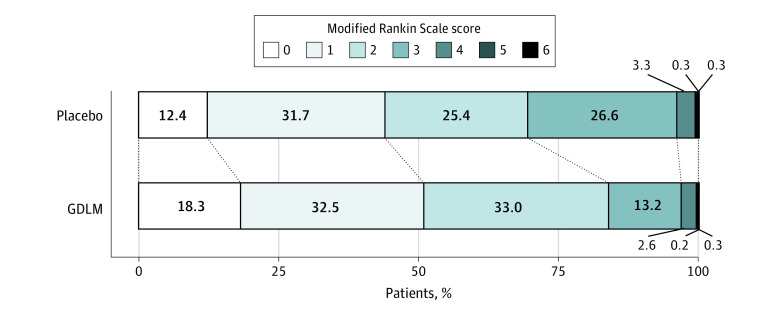
Distribution of 90-Day Modified Rankin Scale (mRS) Score The mRS is a global stroke disability scale with scores ranging from 0 (no symptoms or completely recovered) to 6 (death). Each cell corresponds to a score on the mRS; the width of the cell indicates the proportion of patients with equivalent scores. The percentage of patients in each category is shown within the cell. GDLM indicates ginkgo diterpene lactone meglumine.

In the modified intention-to-treat analysis, 877 (50.8%) of 1725 patients in the GDLM group and 759 (44.1%) of 1723 patients in the placebo group reached the primary outcome (mRS score of 0 or 1 at 90 days; risk difference, 6.79%; 95% CI, 3.46%-10.10%; OR, 1.31; 95% CI, 1.15-1.50; RR, 1.15; 95% CI, 1.08-1.24; *P* < .001; Table 2, Figure 2). The sensitivity analysis yielded similar results (eTable 4 in Supplement 2).

**Table 2.** Efficacy Outcomes

Outcomes	GDLM group (n = 1725)	Placebo group (n = 1723)	Risk difference (95% CI), %	Odds ratio (95% CI)	Relative risk (95% CI)	*P* value
Primary outcome						
mRS score of 0 or 1 at day 90, No. (%)^a^	877 (50.8)	759 (44.1)	6.79 (3.46 to 10.10)	1.31 (1.15 to 1.50)	1.15 (1.08 to 1.24)	<.001
Secondary outcomes						
mRS score of ≤2 at day 90, No. (%)^a^	1446 (83.8)	1197 (69.5)	14.35 (11.56 to 17.12)	2.28 (1.93 to 2.68)	1.21 (1.16 to 1.25)	<.001
NIHSS score^b^ change of ≤−4 from baseline to day 7, No. (%)^c^	461 (27.7)	414 (25.0)	2.70 (−0.29 to 5.69)	1.15 (0.98 to 1.34)	1.11 (0.99 to 1.24)	.08
NIHSS score^b^ change of ≤−4 from baseline to day 14, No. (%)^d^	1000 (61.3)	810 (50.2)	11.13 (7.72 to 14.51)	1.57 (1.37 to 1.81)	1.22 (1.15 to 1.30)	<.001
NIHSS score^b^ change of ≥4 from baseline to day 7, No. (%)^c^	13 (0.8)	11 (0.7)	0.12 (−0.50 to 0.74)	1.18 (0.53 to 2.64)	1.18 (0.53 to 2.62)	.69
NIHSS score^b^ change of ≥3 from baseline to day 7, No. (%)^c^	17 (1.0)	18 (1.1)	−0.07 (−0.80 to 0.66)	0.94 (0.48 to 1.83)	0.94 (0.49 to 1.82)	.85
NIHSS score^b^ change of ≥2 from baseline to day 7, No. (%)^c^	32 (1.9)	35 (2.1)	−0.19 (−1.18 to 0.79)	0.91 (0.56 to 1.47)	0.91 (0.57 to 1.46)	.70
NIHSS score^b^ change of ≥1 from baseline to day 7, No. (%)^c^	50 (3.0)	77 (4.6)	−1.64 (−2.98 to −0.34)	0.64 (0.44 to 0.91)	0.65 (0.46 to 0.92)	.01
Safety outcomes						
Adverse events, No. (%)	303 (17.6)	298 (17.3)	0.27 (−2.26 to 2.80)	1.02 (0.85 to 1.21)	1.02 (0.88 to 1.17)	.83
Severe adverse events, No. (%)	26 (1.5)	22 (1.3)	0.23 (−0.57 to 1.05)	1.18 (0.67 to 2.10)	1.18 (0.67 to 2.07)	.56

Abbreviations: GDLM, ginkgo diterpene lactone meglumine; mRS, modified Rankin Scale; NIHSS, National Institutes of Health Stroke Scale.

^a^ The mRS is a global stroke disability scale with scores ranging from 0 (no symptoms or completely recovered) to 6 (death).

^b^ The NIHSS is a tool used by clinicians to quantify impairment caused by stroke (range, 0-42, with higher scores indicating greater severity). A decrease in score indicates a functional improvement, while an increase in score indicates symptom regression.

^c^ The number of patients with missing data was similar in the 2 treatment groups. Missing data for NIHSS score on day 7 occurred in 61 patients in the GDLM group and 67 patients in the placebo group.

^d^ The number of patients with missing data was similar in the 2 treatment groups. Missing data for NIHSS score on day 14 occurred in 95 patients in the GDLM group and 110 patients in the placebo group.

#### Secondary Outcomes

**Figure 3.**
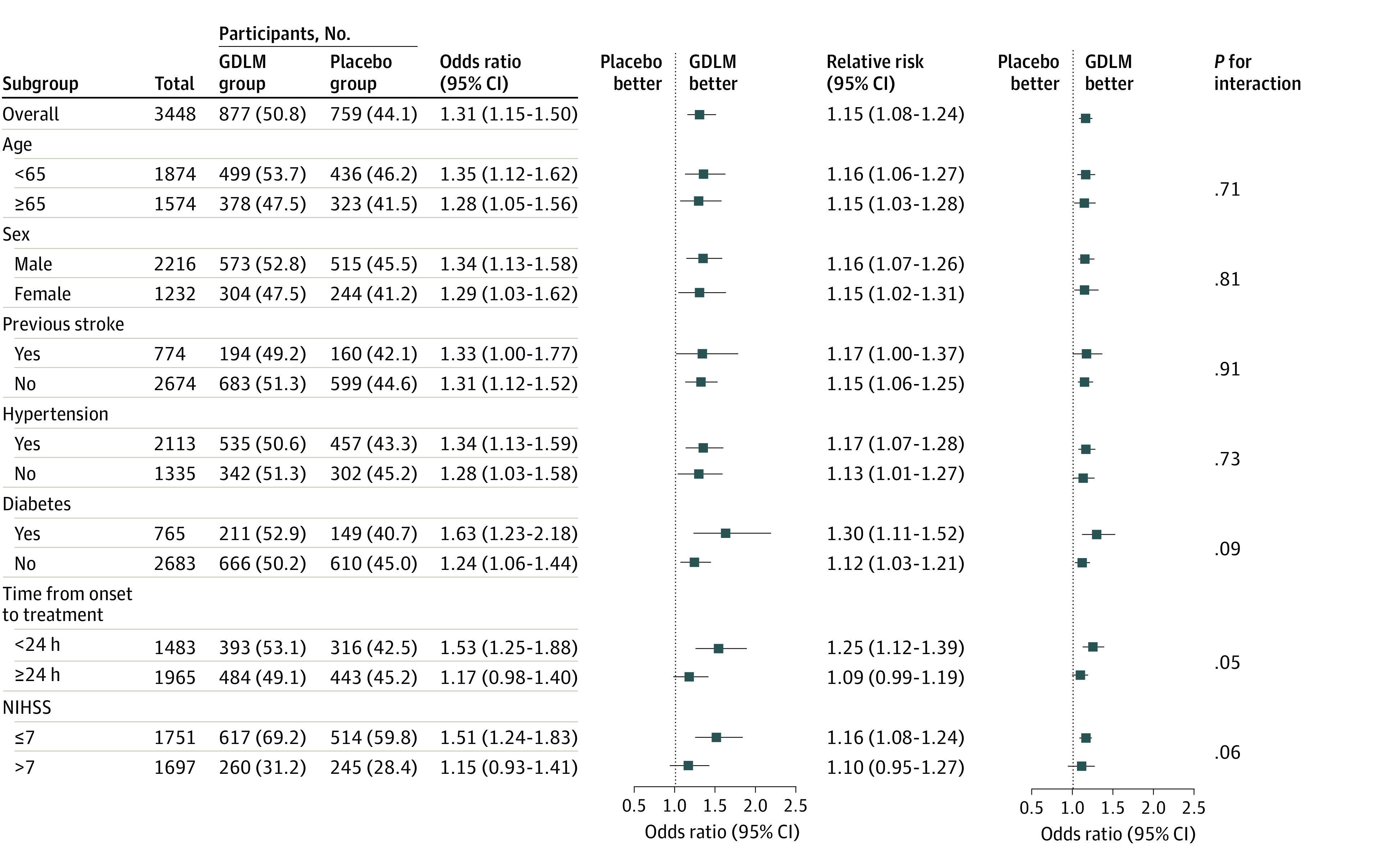
Odds Ratio for the Primary Outcome in Prespecified Subgroups The *P* for interaction value was calculated based on the odds ratios with logistic regression. Abbreviations: GDLM, ginkgo diterpene lactone meglumine; NIHSS, National Institutes of Health Stroke Scale. The NIHSS is a tool used by clinicians to quantify impairment caused by stroke (range, 0-42, with higher scores indicating greater severity).

With respect to secondary outcomes, the proportion of patients with a favorable functional outcome (mRS score of 0-2) in the GDLM group was 83.8% compared with 69.5% in the placebo group (risk difference, 14.35%; 95% CI, 11.56%-17.12%; OR, 2.28; 95% CI, 1.93-2.68; RR, 1.21; 95% CI, 1.16-1.25; *P* < .001). The proportion of patients with a between-group difference in NIHSS scores from baseline to day 14 of at least −4 in the GDLM group was 61.3%, compared with 50.2% in the placebo group (risk difference, 11.13%; 95% CI, 7.72%-14.51%; OR, 1.57; 95% CI, 1.37-1.81; RR, 1.22; 95% CI, 1.15-1.30; *P* < .001). The proportion of patients with a between-group difference in NIHSS score from baseline to day 7 of at least +1 in the GDLM group was 3.0%, compared with 4.6% in the placebo group (risk difference, −1.64%; 95% CI, −2.98% to −0.34%; OR, 0.64; 95% CI, 0.44-0.91; RR, 0.65; 95% CI, 0.46-0.92; *P* = .01). No significant treatment effect was observed in other secondary outcomes. The results of the per-protocol analysis were consistent with those of the modified intention-to-treat analysis (eTable 5 in Supplement 2). Similar efficacy was observed in the prespecified subgroups (Figure 3).

#### Safety Outcomes

The GDLM group and the placebo group had a similar incidence of adverse events (303 [17.6%] vs 298 [17.3%]; risk difference, −0.27%; 95% CI, −2.26%-2.80%; OR, 1.02; 95% CI, 0.85-1.21; RR, 1.02; 95% CI, 0.88-1.17; *P* = .83; Table 2; eTable 6 in Supplement 2), a similar incidence of severe adverse events (26 [1.5%] vs 22 [1.3%]; risk difference, 0.23%; 95% CI −0.57% to 1.05%; OR, 1.18; 95% CI, 0.67-2.10; RR, 1.18; 95% CI, 0.67-2.07; *P* = .56; Table 2; eTable 7 in Supplement 2), and similar vital signs and laboratory examination result values (eTable 8 in Supplement 2).

## Discussion

In this randomized, double-blind, placebo-controlled, parallel-group clinical trial, GDLM dispensed within 48 hours after symptoms for patients with AIS improved the proportion of patients achieving a 90-day favorable functional outcome, which was defined as an mRS score of 0 or 1, compared with placebo.

Neuroprotective agents, which could target cerebral parenchyma in the acute ischemic phase and restore neuronal function in the after-stroke phase, have been widely acknowledged as a promising option for AIS treatment.^17^ However, though numerous preclinical evaluations of neuroprotectants have fostered high expectations of their clinical efficacy, most prior clinical trials targeting neuroprotection failed, showing nonsignificant clinical efficacy for AIS patients.^18^ For instance, magnesium sulfate, which was proven by diverse animal models to have vasodilatory and direct neuroprotective and glioprotective effects, failed to improve 90-day disability outcomes for patients with suspected stroke.^3^ A free radical-trapping agent, NXY-059, was also found to be ineffective for the treatment of AIS by the SAINT (Stroke-Acute Ischemic NXY Treatment) I and II trials.^4^ Similar results were also found for other neuroprotective drugs, including intravenous albumin, natalizumab, and uric acid.^5,6,7^ Possible reasons underlying the failures may include, but are not limited to, the single molecular targeted role of the neuroprotection, the study design, patient characteristics, time to treatment, and quality criteria, as previously summarized.^19^

Recently, the exploratory analysis of the ESCAPE-NA1 (Efficacy and Safety of Nerinetide for the Treatment of Acute Ischemic Stroke) trial indicated a potential efficacy of nerinetide for patients who didn’t receive treatment of alteplase.^20^ More directly, edaravone dexborneol, a novel multitargeted neuroprotectant, was reported to have significant benefits in improving functional outcomes for patients with AIS in the TASTE (Edaravone Dexborneol vs Edaravone Alone for the Treatment of Acute Ischemic Stroke) trial.^21^ These previously mentioned trials indicated, to some extent, that multitargeted neuroprotectants might be more promising for AIS treatment than those targeting a single pathway, partially due to the complex process of ischemic damage.

Ginkgolide-related intravenous preparation is a class of multitargeted neuroprotectants widely used for the treatment of cardiocerebrovascular diseases in China,^22^ of which GDLM was approved for clinical use among patients with ischemic stroke in 2012 (national medicine permission number, Z20120024). To date, a series of randomized clinical trials have been conducted in China to evaluate the efficacy and safety of GDLM, a large portion of which were single-center trials with a limited sample size of approximately 100 to 200 patients. Moreover, most previous randomized clinical trials were not placebo controlled. They commonly made direct comparisons between GDLM and other neuroprotective agents like Danhong, Shuxuening, Deengzhanxixin, and Butylphthalide injections.^11,14^ Therefore, evidence for the single-therapeutic response to GDLM remains scarce, to our knowledge, until now. The present study, which involved a large sample size of more than 3000 participants and used a multicenter, placebo-controlled design with strict quality control, confirmed the potential therapeutic value of GDLM in patients with AIS reported previously. Further, the present study provided more precise and direct estimates of GDLM’s clinical efficacy.

Ginkgo diterpene lactone meglumine has shown a multipathway neuroprotective and reparative effect in preclinical animal trials. For instance, it was reported to exert its neuroprotective effect through antioxidation, increasing the level of antioxidant enzymes, maintaining the dynamic balance of oxygen free radical formation, and restraining the trigger of lipid peroxidation.^12^ In addition, in vitro studies also indicated that it could promote the secretion of brain-derived neurotrophic and ciliary neurotrophic factors, thus regulating the sphingolipid and neurotrophic signaling pathways.^9^ Other potential mechanisms, including anti-inflammation, antiapoptosis, and proangiogenesis, have also been reported.^9,11,12,13^

### Limitations

Several limitations in the present study need to be addressed. First, because the current trial was mainly conducted among patients with Han Chinese backgrounds, caution should be taken when generalizing these findings to other ethnic groups. Additionally, patients with ischemic stroke in this trial did not receive intravenous thrombolysis or mechanical thrombectomy. However, given the increasing popularity of recanalization therapy, further research on the efficacy and safety of GDLM based on successful recanalization is warranted.

## Conclusions

The current randomized clinical trial indicated that among patients with AIS treated within 48 hours after onset, the 14-day treatment of GDLM could improve the proportion of patients achieving good clinical outcomes at 90 days compared with placebo.

## Notes

Supplement 1.
Trial Protocol

Supplement 2. **eTable 1.** Inclusion and Exclusion Criteria **eTable 2.** Concomitant Treatment Within 90 Days **eTable 3.** Concomitant Prohibited Medication Used Within 90 Days **eTable 4.** Sensitivity Analyses **eTable 5.** Primary and Secondary Outcomes in Per-protocol Population **eTable 6.** Adverse Events Recorded During 90 Days of Follow-up **eTable 7.** Severe Adverse Events (by System Organ Class) During 90 Days of Follow-up **eTable 8.** Number of Patients with Clinically Significant Changes in Vital Signs and Laboratory Data on Day 14 of Follow-up

Supplement 3.
Nonauthor Collaborators

Supplement 4.
Data Sharing Statement
