# Supplementary material for: Efficacy and Safety of Ginkgo Diterpene Lactone Meglumine in Acute Ischemic Stroke: A Randomized Clinical Trial
Source: JAMA Netw Open. 2023 Aug 14;6(8):e2328828. doi: 10.1001/jamanetworkopen.2023.28828 (PMC10425831; doi:10.1001/jamanetworkopen.2023.28828)
Supplement: Supplement 1. — Trial Protocol [file jamanetwopen-e2328828-s001.pdf]

This supplement contains the following items:

1. Protocol.

2. Statistical analysis plan.

The Efficacy and Safety of Ginkgo Diterpene Lactone Meglumine in Acute Ischemic Stroke

Protocol

Research team: Beijing Tiantan Hospital, Capital Medical University

Principal Investigator: Yongjun Wang

Protocol version 1.0

Aug 1st, 2015

Catalog

Abstract of research protocol 6

Abbreviations 10

1 Background 12

1.1 Preclinical studies 12
1.2 Results of phase I clinical trial 14
1.3 Results of phase II clinical trial 14
1.4 Phase III clinical study 17
1.5 An overview of known or potential risks and benefits for subjects 19
1.6 Regulations to be followed by this study 20

2 Study objectives 21

2.1 Primary objective 21
2.2 Secondary objectives 21
2.3 Exploratory objectives 21
2.4 Safety objectives 22
2.5 Other objectives 22

3 Study design 23

3.1 Overall design 23
3.2 Multicenter 23
3.3 Randomization 23
3.4 Blinding 23
3.4.1 Blinding design 24
3.4.2 Management and preservation of blind codes 24
3.4.3 Emergency unblinding 24
3.5 Study population 25
3.6 Clinical trial process 25
3.7 Study drug 26
3.7.1 Study drug information 26
3.7.2 Dosage 27
3.7.3 Random blinding of drug 27
3.7.4 Drug storage and allocation 28
3.7.5 Drug dispensing, recalling, and counting 29
3.8 Follow-up time of subjects in the trial 29
3.9 Standards for termination of the trial 29

4 Patient selection 30

4.1 Diagnostic criteria for ischemic stroke 30
4.1.1 Atherosclerotic thrombotic cerebral infarction 30
4.1.2 Cerebral embolism 30
4.1.3 Lacunar infarct 31
4.1.4 Asymptomatic infarction 31

4.2 Inclusion criteria ... 31
4.3 Exclusion criteria ... 32
4.4 Conditions and procedures for patients to withdraw from the study ... 33
4.4.1 Investigator-determined withdrawal ... 33
4.4.2 Subjects voluntarily withdrew from the study ... 33
4.5 Case exclusion criteria ... 34
5 Treatment ... 35
5.1 Dose and treatment protocol ... 35
5.1.1 Medication ... 35
5.1.2 Medication precautions ... 35
5.1.3 Drug incompatibility ... 37
5.2 Medication period ... 37
5.3 Concomitant medication ... 37
5.3.1 Concomitant medication during the study ... 37
5.3.2 Permitted concomitant medication ... 38
5.3.3 Drugs and treatments prohibited during the study ... 39
5.3.4 Rehabilitation training regulations ... 39
5.4 Compliance evaluation ... 40
6 Efficacy evaluation ... 40
6.1 Primary efficacy measure ... 40
6.2 Secondary efficacy measures ... 40
6.3 Exploratory measures ... 41
7 Safety evaluation ... 42
7.1 Safety measures ... 42
7.2 Adverse events ... 42
7.2.1 Definition of adverse events ... 42
7.2.2 Observation and recording of adverse events ... 42
7.2.3 Severity of adverse events ... 43
7.2.4 Correlation of adverse events with drugs ... 43
7.3 Severe adverse events ... 44
7.4 Recording, processing, and reporting ... 45
7.5 Emergency Procedures ... 46
8 Data management and statistical analysis ... 47
8.1 Independent Data and Safety Monitoring Board (DSMB) ... 47
8.2 Data management ... 47
8.3 Statistical analysis ... 49
8.3.1 Sample Size Estimate ... 49
8.3.2 General Principles ... 49
8.3.3 Study Population ... 50
8.3.4 Demographic and Baseline Characteristics ... 51
8.3.5 Efficacy Analyses ... 52
8.3.6 Safety Analysis ... 56

9 Quality control and quality assurance 58
9.1 Quality control 58
9.2 Quality assurance 58
10 Ethical Principles 59
11 Data preservation and conclusion 60
12 Responsibilities assumed by all parties 61
13 References 61
Annex 1: National Institutes of Health Stroke Scale (NIHSS) 63
Annex 2: Modified Rankin Scale (mRS) 75
Annex 3: Montreal Cognitive Assessment Scale (MoCA) 78
Annex 4: EuroQol Five-Dimensions (EQ-5D) Health Scale 86

Abstract of research protocol

**Research Title**	The Efficacy and Safety of Ginkgo Diterpene Lactone Meglumine in Acute Ischemic Stroke
**Research Team**	Beijing Tiantan Hospital, Capital Medical University
**Research Center**	About 120 centers in China
**Indication**	Acute ischemic stroke
**Study Objective**	To evaluate whether ginkgo diterpene lactone meglumine can improve functional outcome (mRS 0-1) on day 90 after randomization compared with placebo in patients with acute ischemic stroke.
**Study design**	A randomized, double-blind, placebo-controlled, multicenter clinical trial.
**Sample size**	A total of 1,726 patients (863 patients in the experimental group and 863 patients in the control group)
**Participants**	**Inclusion criteria:**<br>(1) Aged 18 to 80 years of age;<br>(2) Within 48 hours of stroke onset of ischemic stroke (diagnosis standard by the Chinese medical association of the fourth national conference on cerebrovascular disease);<br>(3) The first onset, or always not obvious legacy of stroke sequela (mRS acuities were before the onset of 1);<br>(4) The degree of nerve function defect score (NIHSS) scores 4 to 24 points, body movement component (NIHSS score paragraphs 5 and 6) total score 2 points or higher;<br>(5) Understand and voluntarily signed informed consent.<br>**Exclusion criteria:**<br>(1) Head imaging studies have confirmed that, encephalitis, brain tumor, brain abscess and cause similar symptoms of disease, or confirm with hemorrhagic cerebral infarction, epidural hematoma,

	intracranial hematoma, intraventricular hemorrhage, subarachnoid hemorrhage, etc.;<br>(2) The serious disturbance of consciousness (Ia NIHSS score 2 points or higher);<br>(3) With hemorrhagic disease or have a bleeding tendency, or have a lower limb venous thrombosis;<br>(4) Serious abnormal liver and kidney function, liver function laboratory indexes of ALT > 3 ULN, renal laboratory ULN Cr > 1.5);<br>(5) A history of mental illness or dementia patients;<br>(6) Severe organ or other systemic disease, accompanied by any organ or system of malignant tumor, or ongoing anti-tumor treatment, the estimated lifetime < 3 months;<br>(7) Significant drug or alcohol abuse;<br>(8) Allergic constitution, as well as to two or more drugs or food allergies; This medicine ingredients allergy or known;<br>(9) Have pregnancy (check blood HCG positive screening tests, namely HCG > 5 miu/mL), during the test preparation is pregnancy or lactation in women;<br>(10) In the past three months in other clinical trials;<br>(11) Researchers do not determine poor adherence, or any other suitable for patients to participate in this study.
**Drugs**	**Experimental drug:**<br>✧ Ginkgo diterpene lactone meglumine injection, 5 ml per bottle (containing 25 mg ginkgo diterpene lactone);<br>✧ Manufacturer: Jiangsu Kangyuan Pharmaceutical Co., LTD.<br>**Control drug:**<br>✧ Ginkgo diterpene lactone meglumine injection simulation, 5 ml per bottle;<br>✧ Manufacturer: Jiangsu Kangyuan Pharmaceutical Co., LTD.

**Treatment protocol**	The study drug was given within 48 hours of onset and patients are randomized to two groups according to a 1:1 ratio.<br>**Intervention group:**<br>Ginkgo diterpene lactone meglumine injection of dosing of 5 ml per time, and 1 time per day. The ginkgo diterpene lactone meglumine injection (25mg) was diluted in 250 mL of sterile 0.9% sodium chloride injection.<br>**Control group:**<br>Ginkgo diterpene lactone meglumine injection simulation of dosing of 5 ml per time, and 1 time per day. The ginkgo diterpene lactone meglumine injection simulation was diluted in 250 mL of sterile 0.9% sodium chloride injection.
**Randomization / Blinding**	✧ Patients will be randomly assigned to ginkgo diterpene lactone meglumine or placebo according to a ratio of 1:1. The block randomization method was adopted to generate the random number table.<br>✧ Ginkgo diterpene lactone meglumine and the placebo were visually identical and cannot be distinguishable by smell. To ensure the implementation of the blind method, physicians, or nurses involved in drug management should not participate in activities such as subject selection, evaluation of efficacy and safety, etc., to avoid introducing bias.
**Methods of administration**	Ginkgo diterpene lactone meglumine/placebo on days 1 to 14.
**Follow-up schedule**	Follow-up at days 1, 7, 14 and 90.
**Efficacy measures**	**Primary efficacy measures:**<br>The proportion of mRS of 0-1 on day 90 after randomization.<br>mRS scores range from 0 to 6, and score 0 indicates no symptoms, score 1 indicates no clinically significant disability, score 2 indicates slight

	disability, score 3 indicates moderate disability, score 4 indicates moderately severe disability, score 5 indicates severe disability, and score 6 indicates death.<br>**Second efficacy measures:**<br>(1) The proportion of patients with mRS 0-2 on day 90;<br>(2) The proportion of patients with recovery of neurological deficits on day 7: decline in NIHSS score ≥4;<br>(3) The proportion of patients with recovery of neurological deficits on day 14: decline in NIHSS score ≥4;<br>(4) The proportion of patients with deterioration of neurological deficits on day 7: increase in NIHSS score increase in NIHSS score ≥4;<br>(5) The proportion of patients with deterioration of neurological deficits on day 7: increase in NIHSS score increase in NIHSS score ≥3;<br>(6) The proportion of patients with deterioration of neurological deficits on day 7: increase in NIHSS score increase in NIHSS score ≥2;<br>(7) The proportion of patients with deterioration of neurological deficits on day 7: increase in NIHSS score increase in NIHSS score ≥1.
**Safety measures**	(1) Adverse events within 90 days;<br>(2) Severe Adverse events within 90 days;<br>(3) Vital signs and laboratory variables from baseline to day 14 after randomization, including blood pressure, heart rate, breathing, body temperature, blood routine (hemoglobin, platelet count, red blood cell, white blood cell count), urine routine (red blood cell count, white blood cell count, glucose, protein), and blood biochemistry (alkaline phosphatase, alanine aminotransferase, aspartate transaminase, γ-glutamyl transpeptidase, total bilirubin, urea nitrogen, creatinine).
**Study duration**	February 2016 - May 2018

Abbreviations

AE	Adverse Event
ALT	Alanine Aminotransferase
CI	Confidence Interval
Cr	Creatinine
CRF	Case Report Form
DRQ	Data Rating Questionnaire
DSMB	Data And Safety Monitoring Board
DVP	Data Validation Plan
EEG	Electroencephalogram
EQ-5D	Euroqol Five-Dimensions Health Scale
FAS	Full Analysis Set
GCP	Good Clinical Practice
GGT	Gamma-Glutamyl Transpeptidase
GSH	Glutathione
HCG	Human Chorionic Gonadotropin
HGB	Hemoglobin
IRB	Institutional Review Board
LOC	Level of Consciousness
LOCF	Last Observation Carry-Forward
MCAO	Middle Cerebral Artery Occlusion
MoCA	Montreal Cognitive Assessment Scale
mRS	Modified Rankin Scale
NIHSS	National Institute of Health Stroke Score
OR	Odds Ratio
PPS	Per Protocol Set
PVC	Polyvinyl Chloride
SAE	Severe Adverse Event

SOD	Superoxide Dismutase
SOP	Standard Operating Procedures
SS	Safety Set
TCM	Traditional Chinese Medicine
TIA	Transient Ischemic Attacks
TMB	Transient Monocular Blindness
ULN	Upper Limits of Normal

1 Background

Ginkgo diterpene lactone meglumine was jointly developed by China Pharmaceutical University and Jiangsu Kangyuan Pharmaceutical co., LTD., and was approved by the China Food and Drug Administration in 2012. The national drug approval number was Z20120024. Its active pharmaceutical ingredients include ginkgolide A, ginkgolide B, and ginkgolide K, etc. The excipients are meglumine, citric acid and sodium chloride. The medicine has the functions of promoting blood circulation and dredging the channels and collaterals. It is used for the syndrome of intermingled phlegm and blood stasis in the recovery period of meridians and collaterals in stroke (mild to moderate cerebral infarction). The symptoms include hemiplegia, crooked tongue, dysphasia, numbness of the limbs, etc.

1.1 Preclinical studies

- ✧ Main pharmacodynamics

Ginkgo diterpene lactone meglumine (7.5, 15.0, 30.0 mg/kg) intravenous injection can reduce stroke score, infarct area, and the water content of brain tissue in the middle cerebral artery occlusion (MCAO) model of rats. There was no significant difference in effect intensity from Jinnado injection. It can reduce the contents of MDA and LA in the brain tissue of focal cerebral ischemia rats with reperfusion injury, and meanwhile increase the contents of SOD and GSH, suggesting that the drug can significantly inhibit the degree of ischemia hypoxia and peroxidation of brain tissue, and improve the antioxidant capacity and the ability to scavenge free radicals. It can obviously protect the brain tissue structure of focal cerebral ischemia rats with reperfusion injury, and significantly reduce the degree of nuclear pyknosis and nuclear dissolution of cerebral cortical pyramidal cells and brain parenchymal nerve cells compared with the ischemic control group, and reduce the softening lesion. It can significantly short the recovery time of EEG and righting reflex in rats with diffuse global cerebral ischemia. It can significantly prolong the survival time of rats under hypoxia, significantly inhibit platelet aggregation and have depolymerization,

significantly inhibit the formation of arteriovenous bypass thrombosis in rabbits and have thrombolytic effect, and significantly prolong the coagulation time.

✧ General pharmacology

Ginkgo diterpene lactone meglumine had no significant effect on the behavior and coordinated movement of mice within the effective dose range, had no synergistic effect on subthreshold dose of pentobarbital sodium, and had no significant effects on blood pressure, heart rate, electrocardiogram, respiratory rate, and amplitude in anesthetized dogs.

✧ Animal acute toxicity experiment

The maximum volume of intravenous injection was 200 mg/kg. Seven days after administration, the animals were in good health, with smooth fur and normal autonomic activity, and no death occurred. Therefore, the LD50 (95% credible limit) of intravenous injection in mice was greater than 200 mg/kg, which was equivalent to 120 times of the daily clinical dosage of adults (100 mg/kg) in terms of kilogram the body weight. Postmortem examination of the mice showed no obvious abnormality.

✧ Animal long-term acute toxicity experiment

The long-term toxicity experiment was conducted in rats. SD rats were administered intraperitoneal injection continuously for 8 weeks at doses of 24, 60, and 150 mg/kg. The results showed the ginkgo diterpene lactone meglumine had no significant effects on the growth, activity, mental state, body weight, and food intake of the rats.

The long-term toxicity experiment was also conducted in dogs. Beagle dogs were administered intravenous injection continuously for 9 weeks at doses of 10, 20 and 40 mg/kg. No death or other abnormal reactions were observed. The results showed the ginkgo diterpene lactone meglumine had no significant effects on the movement, body weight and food intake of the dogs.

✧ Safety experiment

The preparation safety experiment of ginkgo diterpene lactone meglumine was

carried out. The results showed that ginkgo diterpene lactone meglumine had no hemolysis and erythrocyte agglutination effects on rabbit blood, and no allergic reaction in healthy guinea pigs. There was no obvious vascular stimulation reaction after ear vein injection, and no pyrogen reaction in rabbits. The above experiment results showed that the preparation was safe.

1.2 Results of phase I clinical trial

The dose of 50 mg might cause adverse events such as dizziness, lightheadness, sleepiness, and increased sleep. It had no significant effect on laboratory indicators of subjects. Therefore, the dose of 50 mg was recommended for phase II clinical trials. The dose of 100 mg could further cause adverse events such as abnormal coordinated movement and excessive urination volume. It had no significant effect on the laboratory indicators of subjects. The risk was not too great, so it could be applied. Although the dose of 140 mg did not cause significant abnormalities in laboratory indicators, adverse events occurred more frequently, so it should be used with caution.

These adverse events (including dizziness, lightheadness, sleepiness, increased sleep, abnormal coordinated movement, and excessive urination volume) were speculated to be related to psychological factors, drugs dilated blood vessels, increased blood flow or steal phenomenon. Symptoms could be reduced or disappear by slowing down the drip rate, and all self-resolved without treatment. The dose of 100 mg may increase the velocity of cerebral blood flow, increase cerebral blood flow volume, and reduce peripheral cerebral vascular resistance, thus demonstrating a certain role in regulating the function of cerebral vascular circulation.

1.3 Results of phase II clinical trial

A randomized, blind, placebo-controlled, multicenter clinical trial was conducted. Patients were assigned at a 1:1:1:1 ratio to receive three doses of ginkgo diterpene lactone meglumine (25 mg, 50 mg, 75 mg) and Shuxuening for 14 days. There were 70, 67, 68, and 68 cases enrolled and randomized in the trial, respectively; there were 2, 3, 1, and 1 case dropped out, respectively; 2 cases were excluded (all from

low-dose group), and 66, 64, 67 and 67 cases completed the trial, respectively.

The results of the influence on neurological deficit were shown as blow. Firstly, there were statistically significant improvement of facial paralysis in low-, medium-, and high- dose groups, improvement of language in low- and high- dose groups, and improvement of shoulder and arm movement, hand movement, lower limb movement, and walking ability in four groups after 7 days of medication compared with before medication (P <0.05). Secondly, there were statistically significant improvement of horizontal staring function in control group, and improvement of facial paralysis, language, shoulder and arm movement, hand movement, lower limb movement, and walking ability in four groups (P <0.05) after 14 days of medication compared with before medication (P <0.05). Thirdly, the order for the decreased value of neurological deficit score from baseline and after 7 days of medication was low-dose group > high-dose group > medium-dose group > control group. The order for the decreased value of neurological deficit score from baseline and after 14 days of medication was high-dose group > low-dose group > medium-dose group > control group. However, there was no statistical significance between these groups (P >0.05).

The results of the influence on Traditional Chinese Medicine (TCM) syndrome were shown as blow. Firstly, there were statistically significant improvement of lower limb paralysis and hypoesthesia in low- and high- dose groups and control group, and improvement of upper limb paralysis, crooked tongue, dysphasia in four groups after 7 days of medication compared with before medication (P <0.05). Secondly, there were statistically significant improvement of main symptoms in four groups after 14 days of medication compared with before medication (P <0.01). Thirdly, the order for the decreased value of TCM syndrome score from baseline and after 7 or 14 days of medication was high-dose group > low-dose group > medium-dose group > control group. There was statistically significant between these groups after 14 days of medication (P<0.05).

The order for the decreased value of the life ability score from baseline and after

7 days of medication was medium-dose group > high-dose group > low-dose group > control group. The order for the decreased value of the life ability score from baseline and after 14 days of medication was high-dose group > medium-dose group > low-dose group > control group. However, there was no statistical significance between these groups (*P*>0.05).

There were statistically significant differences in whole blood viscosity (high cut) in high-dose group, and in whole blood viscosity (low cut) and plasma viscosity in low-dose, medium-dose and high-dose groups after medication compared with before medication (*P*<0.05).

The total efficacy of disease was high-dose group > low-dose group > medium-dose group > control group (*P*<0.05). The total efficacy of TCM syndromes was high-dose group > low-dose group > medium-dose group > control group (*P*>0.05).

For safety evaluation, 2 patients (2.90%) in the low-dose group, 7 patients (10.45%) in the medium-dose group, 2 patients (2.99%) in the high-dose group, and 2 patients (2.99%) in the control group occurred adverse events. Adverse reactions including fever, chills, dizziness occurred in 3 patients in the control group and 0 patients in the other groups. One severe adverse event occurred in the experimental group, which was death due to pulmonary embolism. There was no evidence that it was related to the drug.

In conclusion, all the 4 groups were safe and effective in treating the syndrome of intermingled phlegm and blood stasis in the recovery period of atherosclerotic thrombotic cerebral infarction. The general trend of efficacy was high-dose group > low-dose group > medium-dose group > control group. There were more adverse events in the medium-dose group (50 mg group), and there was no statistically significant difference in efficacy between the low-dose group (25 mg group) and the high-dose group (75 mg group) (*P*>0.05). Therefore, the recommended dose for phase III clinical trial was 25 mg per time, one time a day.

1.4 Phase III clinical study

Phase III clinical trial was a multicenter, randomized, blind, positive, parallel controlled trial. The ginkgo diterpene lactone meglumine was used in the experimental group and the Shuxuening was used in the control group. 621 participants were enrolled in the study, of which 413 with the cerebral infarction (309 assigned to the experimental group, of them, 307 completed, 1 dropped out, and 1 removed; 104 assigned to the control group, of them, 101 completed, 3 dropped out, and 1 removed) and 208 with the lacunar infarction (104 assigned to the experimental group, of them, 104 completed; 104 assigned to the control group, of them, 102 completed, 1 dropped out, and 1 removed). Only safety evaluation but not efficacy evaluation was performed for patients with lacunar infarction. Results of the trial were shown as follow.

For total efficacy, the efficacy rate (basic recovery or significant improvement) of the experimental group was 48.05%; The efficacy rate of the control group (basic recovery or significant improvement) was 24.75%. There was a statistically significant difference between the two groups (P<0.01). The total efficacy rate (basic recovery or significant improvement or improvement) of the experimental group and the control group was 85.39% and 73.27%, respectively, and the difference between the two groups was statistically significant (P<0.01).

For influence on neurological deficit, there were statistically significant differences in the total score of neurological deficits before medication and that on 7 days after medication, and before and after medication (P<0.01). There was no statistically significant difference in the total score of neurological deficits between the experimental and control group (P>0.05). Similarly, there were statistically significant differences in the decrease rate of the total score of neurological deficits between before medication and 7 days after medication, and between before and after medication (P<0.01). The difference between groups was statistically significant considering the central effect (P<0.01). In addition, there were statistically significant

differences in facial paralysis, language, shoulder and arm movement, hand movement, lower limb movement, and walking ability between the two groups before and after medication (*P*<0.01), while there was only a statistically significant difference in horizontal staring function between the two groups (*P*<0.01).

For influence on life ability status, there were statistically significant differences in the score of life ability status before and after medication in the experimental group and that of the control group (*P*<0.01). There was no significant difference in the score of life ability status between the two groups (*P*>0.05).

For influence on TCM syndrome, the effective rate of the experimental group and the control group was 7.79% and 3.96% respectively, and the total effective rate was 62.99% and 40.59% respectively, the difference of which was statistically significant (*P*<0.01). The differences in decrease rate before and after medication of the scores of TCM syndromes were statistically significant in the experimental group and that of the control group (*P*<0.01). There were statistically significant differences in decrease rate before and after medication between the experimental and control group on 7 days after medication and 14 days after medication (*P*<0.01). For the experimental group and control group, there were statistically significant differences in main TCM syndromes including upper limb paralysis, lower limb paralysis, twisted mouth, fluency disorder, aphasia, hypoesthesia, anesthesia, dizziness, and phlegm before medication and that on day 14 after medication (*P*<0.01). Differences between the experimental group and control group were analyzed before and after medication, there were statistically significant differences in upper limb paralysis, twisted mouth, dizziness, and phlegm (*P*<0.05, *P*<0.01), and no statistically significant difference in other symptoms (*P*>0.05). The disappearance rates of dull tongue quality, white tongue coating greasy, and pulse string slippery were compared between experimental and control groups before and after medication, only the differences in white tongue greasy and pulse string slippery were statistically significant (*P*<0.05), the other differences were not statistically significant (*P*>0.05).

For influence on hemorheology, there was no statistically significant difference in the whole blood viscosity (high cut), whole blood viscosity (low cut), and plasma viscosity before and after medication in the experimental group and that of the control group (P>0.05), and there was no statistically significant difference between the two groups (P>0.05).

The incidence of adverse events in patients with cerebral infarction in phase III trial was 3.90% in the experimental group and 0.99% in the control group. The incidence of adverse reactions was 0.65% in the experimental group and 0 in the control group, mainly manifested as abnormal liver function and facial rash. No severe adverse events were reported in either group. The incidence of adverse events in phase III clinical trial of patients with lumen infarction was 4.81% in the experimental group and 0.97% in the control group. The incidence of adverse reactions in the experimental group and control group was 0. No severe adverse events were reported in either group.

The results of phase III trial showed that ginkgo diterpene lactone meglumine had the effects of phlegm reducing, pulse clearing, and collaterals soothing, and it was safe and efficacious for the treatment of phlegm-stasis blocking collaterals syndrome in the convalescent period of atherosclerotic thrombotic cerebral infarction. It was in good safety for the treatment of phlegm-stasis blocking collaterals syndrome in the convalescent period of lacunar cerebral infarction.

1.5 An overview of known or potential risks and benefits for subjects

The adverse reactions in the product instructions are described as follows:

(1) Some patients showed dizziness, headache, back pain, neck pain, increased urination, increased nocturia, tiredness, drowsiness, coordination function disorder, etc.

(2) A few patients showed shiver, fever, palpitation, posterior occipital discomfort, mild cyanosis of lips and claws, shaking of lower limbs, diarrhea, etc. If

the above symptoms occur, the medication should be stopped immediately and patients should be treated accordingly.

(3) Some patients showed allergic reactions such as red spot rash after medication.

(4) ALT and AST increased in a few patients after medication.

(5) Some patients may suffer blood pressure instability during medication, mainly manifested as blood pressure reduction.

The adverse reactions observed in preliminary clinical trials included fever (0.16%), shivering (0.33%), dizziness (0.16%), facial rash (0.16%), liver function deficit (0.16%), etc.

According to literature review on adverse reactions of other ginkgoales intravenous administration preparation, combined with other common adverse reactions of TCM injections, participants may also suffer digestive system reactions (nausea, vomiting, diarrhea, etc.), respiratory system reactions (shortness of breath, breathlessness, dyspnea, allergic asthma, etc.), nervous system reactions (headache, dizziness or aggravation, etc.), systemic reactions (fever, shivering), other system reactions (phlebitis, blurred vision), and unexpected adverse reactions such as local reactions, laryngeal edema, pyrogen reaction, anaphylactic shock or even death, etc.

After the clinical trials of phase I, phase II, phase III, and the clinical application after the marketing, it can be shown that ginkgo diterpene lactone meglumine can improve the efficacy of disease, neurological deficit, living ability, and TCM syndrome in patients with ischemic stroke.

1.6 Regulations to be followed by this study

This trial will be carried out following the relevant provisions of the 2003 edition of the "Good Practice for Quality Management of Drug Clinical Trials".

2 Study objectives

2.1 Primary objective

To evaluate whether ginkgo diterpene lactone meglumine can improve functional outcome (mRS 0-1) on day 90 after randomization compared with placebo in patients with acute ischemic stroke.

2.2 Secondary objectives

- ✧ To evaluate the difference in the proportion of mRS 0-2 on day 90 after randomization between the two groups;
- ✧ To evaluate the difference in the recovery of neurological deficits (decline in NIHSS score ≥ 4) on day 7 between the two groups;
- ✧ To evaluate the difference in the recovery of neurological deficits (decline in NIHSS score ≥ 4) on day 14 between the two groups;
- ✧ To evaluate the difference in deterioration of neurological deficits (increase in NIHSS score ≥ 4) on day 7 between the two groups;
- ✧ To evaluate the difference in deterioration of neurological deficits (increase in NIHSS score ≥ 3) on day 7 between the two groups;
- ✧ To evaluate the difference in deterioration of neurological deficits (increase in NIHSS score ≥ 2) on day 7 between the two groups;
- ✧ To evaluate the difference in deterioration of neurological deficits (increase in NIHSS score ≥ 1) on day 7 between the two groups.

2.3 Exploratory objectives

- ✧ To evaluate the difference in the improvement of MoCA from baseline to day 14 between the two groups;
- ✧ To evaluate the difference in the improvement of MoCA from baseline to day 90 between the two groups;

✧ To evaluate the difference in the improvement of EQ-5D from baseline to day 14 between the two groups;

✧ To evaluate the difference in the improvement of EQ-5D from baseline to day 90 between the two groups;

✧ To evaluate the difference in the rate of all-cause mortality within 90 days between the two groups;

✧ To evaluate the difference in the rate of recurrent ischemic stroke within 90 days between the two groups;

✧ To evaluate the difference in the rate of combined vascular event (including stroke, myocardial infarction, vascular death, and angina pectoris) within 90 days between the two groups.

2.4 Safety objectives

✧ To evaluate the difference in the adverse events (AEs) within 90 days between the two groups;

✧ To evaluate the difference in the severe adverse events (SAEs) within 90 days between the two groups;

✧ To evaluate the difference in the changes of vital signs and laboratory test values from baseline to day 14 after randomization between the two groups. The variables include blood pressure, heart rate, breathing, body temperature, blood routine (hemoglobin, platelet count, red blood cell, white blood cell count), urine routine (red blood cell count, white blood cell count, glucose, protein), and blood biochemistry (alkaline phosphatase, alanine aminotransferase, aspartate transaminase, γ-glutamyl transpeptidase, total bilirubin, urea nitrogen, creatinine).

2.5 Other objectives

Subgroup analyses: the primary efficacy outcome will also be analyzed stratified by age (<65/≥65), sex (male/female), previous stroke (yes/no), hypertension (yes/no),

diabetes mellitus (yes/no), time from onset to treatment (<24/≥24), and NIHSS score (≤7/>7).

3 Study design

3.1 Overall design

This is a randomized, double-blind, placebo-controlled, multicenter clinical trial.

3.2 Multicenter

This study was conducted in about 120 centers. Beijing Tiantan Hospital affiliated with Capital Medical University was responsible for the study. Beijing Shijitan Hospital affiliated with Capital Medical University, Zhongshan Hospital Affiliated with Fudan University, the First Affiliated Hospital of the Fourth Military Medical University (Xijing Hospital), Tianjin Medical University General Hospital, the Second Hospital of Hebei Medical University, Gulou Hospital Affiliated to Nanjing University Medical School, and the First Hospital of Jilin University participated in the study.

3.3 Randomization

Patients will be randomly assigned to ginkgo diterpene lactone meglumine or placebo according to a ratio of 1:1. The block randomization method was adopted to generate the random number table, which was provided by statistics professionals in the Department of Health Statistics of the Fourth Military Medical University using SAS 9.4 software. Personnel unrelated to this trial will complete the preparation of drug blinding and emergency letters.

3.4 Blinding

Ginkgo diterpene lactone meglumine and the placebo were visually identical and cannot be distinguishable by smell. To ensure the implementation of the blind method,

physicians, or nurses involved in drug management should not participate in activities such as subject selection, evaluation of efficacy and safety, etc., to avoid introducing bias.

3.4.1 Blinding design

A two-level blinding design was adopted in this trail. The first level is the group corresponding to each subject (e.g., group A, group B), and the second level is the treatment corresponding to each group (e.g., experimental drug, placebo).

3.4.2 Management and preservation of blind codes

The random number table was generated by the department of clinical data management and statistical analysis. The blind code was sealed in duplicate and kept by the clinical trial responsible unit and the sponsor. The entire process of drug coding will be documented by the person who codes blind, that is, the blinding record, which is kept as one of the documents of the clinical trial. The emergency envelopes will be kept by the principal investigator at each trial center and placed in a place known to all the researchers. No one is allowed to open the envelope without authorization.

3.4.3 Emergency unblinding

When an emergency (such as a SAE) occurs during the trial, or if the patient needs to be rescued and must know what kind of treatment the patient has received, the blind can be broken urgently. Emergency blinding breaking must be decided by the principal investigator at each trial center, and the reason, time, and place of blind breaking must be recorded in detail, and signed. Inform the trial leader hospital and clinical research assistant in time after blind breaking. Data of these subjects should be completely kept. The emergency letter will be sent to each clinical trial center with the corresponding coded drugs and will be withdrawn uniformly after the end of the trial.

3.5 Study population

Patients with acute ischemic stroke who meet inclusion/exclusion criteria will be included in this trial, with 1,726 patients in the experimental group and 1,726 patients in the placebo group, which will be conducted at approximately 120 centers in China.

3.6 Clinical trial process

Research stage		Screening	Following		
		Visit 1	Visit 2	Visit 3	Visit 4
Visits		Randomization Day 0	Day 7 ±1	Day 14±2	Day 90±7
General condition	Informed consent signed	▲			
	Demographic characteristics [1]	▲			
	Current medical history collection [2]	▲			
	Vital sign [3]	▲	▲	▲	
Inclusion/Exclusion	Inclusion/Exclusion	▲			
Laboratory tests	Blood routine	▲	▲	▲	
	Urine routine	▲	▲	▲	
	Blood biochemistry	▲	▲	▲	
	ECG	▲	▲	▲	
	Screening index [4]	▲			
Scores	NIHSS	▲	▲	▲	
	mRS	▲			▲
	MoCA	▲		▲	▲
	EQ-5D	▲		▲	▲
Others	AE/SAEs		▲	▲	▲
	Recurrent ischemic stroke		▲	▲	▲

	Combined vascular event		▲	▲	▲
	Death		▲	▲	▲
	Concomitant treatment	▲	▲	▲	▲
	Randomization and assign drug number	▲			
	Compliance			▲	

Notes:

1. Demographic characteristics includes age, gender, height, weight, ethnicity, marital status, occupation, etc.

2. Medical history information includes past and current histories of disease, allergy history, co-morbidity, and medications, etc.

3. Vital signs were measured after the patient rested quietly for 10 minutes.

4. The screening indicators include pregnancy test and cranial imaging. The pregnancy test is only indicated for women of childbearing age (not menopausal or less than 12 months from menopause).

3.7 Study drug

3.7.1 Study drug information

(1) Experimental group

Ginkgo diterpene lactone meglumine injection, 5 ml per bottle (containing 25 mg ginkgo diterpene lactone), manufactured by Jiangsu Kangyuan Pharmaceutical Co., LTD. Validity: 18 months.

(2) Placebo group

Ginkgo diterpene lactone meglumine injection simulation, 5 ml per bottle, manufactured by Jiangsu Kangyuan Pharmaceutical Co., LTD.

All study drugs were check-qualified.

3.7.2 Dosage

(1) Experimental group

Ginkgo diterpene lactone meglumine injection, 5 ml per time, one time a day.

(2) Placebo group

Ginkgo diterpene lactone meglumine injection simulation, 5 ml per time, one time a day.

3.7.3 Random blinding of drug

(1) Drug preparation

First, random sampling is carried out among the drugs prepared by the sponsor (experimental drug, placebo), and the corresponding drug test reports are provided. Secondly, the drugs should be packaged by the sponsor according to the drug packaging requirements.

(2) Preparation of emergency letter

Emergency letters for the clinical trial, drug number, and the regulations for unblinding in case of emergency are printed on sealed and opaque envelopes. If disassembled, please indicate who dismantled, dismantled date, reason, etc., and record it in the case report form.

The subject's medication information and the institution and address that should be reported immediately are printed inside the envelope. After the emergency letter is prepared, it will be sent to each center along with the drug, and will be collected after the end of the study.

(3) Drug packaged and coded according to the random number table

Personnel not related to the clinical observation, monitoring, and statistical analysis, etc. of the trial will paste the corresponding drug number on the prominent position of the external package of the drug according to the formed processing code.

(4) Random number table and blinding record

The entire process of drug coding will be documented by the person who codes blind, that is, the blinding record, which is kept as one of the documents of the clinical trial. The contents include the preparation of the sponsor's drugs, drug packaging, usage, requirements for storage, drug distribution, generation of the random number, medicine boxes packed by each subject, emergency letters, testing reports of experimental drugs and placebos, preservation of blind bottoming, regulations on unblinding, and the number of medicine boxes distributed to each center, etc.

(5) Distribution of packaged drug

The packaged drugs will be sent to each center according to the random center number, together with the emergency letter of the corresponding drug number.

3.7.4 Drug storage and allocation

Drug administrators should dispense drugs in drug number order and should not select drugs. The drug number shall remain the same throughout the study. The researchers should provide each subject with sufficient study drugs with the same drug number in the order of enrollment, and each subject's drugs were kept and delivered by a fixed doctor or nurse.

Experimental drugs and placebo drugs should be stored in a cold place (0-10℃) away from light.

Drug administrators should fill out the “Study Drug Use Record Form” timely. Emergency methods and measures of the drug shall be provided by the sponsor and kept with the principal investigators of each trial site.

To ensure the implementation of the blinding, doctors or nurses involved in drug management should not participate in subjects’ selection, the evaluation of efficacy and safety, to avoid introducing bias.

3.7.5 Drug dispensing, recalling, and counting

According to the random drug number assigned by each center, the corresponding drugs were dispensed to each center. Patients will be screened in each center, qualified person will be included, and the drug administrator will distribute drugs one by one according to the drug number of each subject (note: only those who meet the inclusion criteria can be dispensed drugs). The drug administrator should fill in the “Study Drug Use Record Form” in time.

During the return visit, the physicians should faithfully record the amounts of drugs used by the subjects, judge the subject's medication compliance, and timely record and sign the “Study Drug Use Record Form”.

3.8 Follow-up time of subjects in the trial

Subjects will be followed up for four times, at 0 day of the screening period (V1), 7±1 day after randomization (V2), 14±2 days after randomization (V3), and 90±7 days after randomization (V4).

3.9 Standards for termination of the trial

Trial termination means the discontinuation of a clinical trial prior to the planned end date. The main purpose of trial termination is to protect subjects' rights and interests, ensure study quality, and avoid unnecessary economic losses.

- ✧ If serious safety problems occur during the trial, the trial should be discontinued in time.
- ✧ If it was found that the effect of drug therapy was too poor, or even ineffective, to be of no clinical value during the trial, the trial should be discontinued to avoid delay of effective treatment for subjects and unnecessary economic losses at the same time.
- ✧ Major errors are found in the study protocol, which would make it difficult to evaluate the efficacy of the drug; or vital protocol deviations occur in the

implementation of a well-designed trial, and it is difficult to evaluate the efficacy of the drug any longer.

- ✧ The sponsor requests discontinuation of the trial (such as reasons for funding and management, etc.).
- ✧ The administrative department cancels the study, etc.

4 Patient selection

4.1 Diagnostic criteria for ischemic stroke

Ischemic stroke was diagnosed using the criteria of the 4th National Academic Conference on Cerebrovascular Diseases of the Chinese Medical Association.

4.1.1 Atherosclerotic thrombotic cerebral infarction

✧ It often develops in a quiet state.

✧ Most of them have no obvious headache and vomiting symptoms at the onset.

✧ The onset is slow, mostly progressive or in stages, mostly related to cerebral atherosclerosis, but also seen in arteritis, blood disorders, etc.

✧ Consciousness is usually clear or mildly impaired within 1 to 2 days after the onset of the disease.

✧ Signs and symptoms of the internal carotid artery system and/or vertebrobasilar system are present.

✧ CT or MRI examination should be performed.

✧ Lumbar puncture cerebrospinal fluid should generally not contain blood.

4.1.2 Cerebral embolism

✧ Most of them have an acute onset.

✧ Most have no prodromal symptoms.

✧ Generally conscious or with transient impairment of consciousness.

✧ Signs and symptoms of the internal carotid artery system and/or vertebrobasilar system are present.

✧ Lumbar puncture cerebrospinal fluid should generally not contain blood, and hemorrhagic cerebral infarction can be considered if there are red blood cells.

✧ The origin of the thrombus can be classified as cardiogenic or non-cardiogenic, and can also be accompanied by embolic symptoms of other organs, skin, and mucosa.

4.1.3 Lacunar infarct

✧ The onset is mostly due to hypertensive atherosclerosis, with acute or subacute onset.

✧ There is mostly no impairment of consciousness.

✧ CT or MRI examination should be performed to clarify the diagnosis.

✧ None of the clinical manifestations are severe. The more common ones are pure sensory stroke, pure motor mild hemiparesis, ataxic mild hemiparesis, dysarthria-clumsy hand syndrome, or sensorimotor stroke, etc.

✧ The lumbar puncture cerebrospinal fluid is free of red blood cells.

4.1.4 Asymptomatic infarction

It is a vascular disease without any cerebral or retinal symptoms, confirmed by imaging only, and may be used as a clinical diagnosis on a case-by-case basis.

4.2 Inclusion criteria

(1) Aged 18 to 80 years of age

(2) Within 48 hours of stroke onset of ischemic stroke (diagnosis standard by the

Chinese medical association of the fourth national conference on cerebrovascular disease);

(3) The first onset, or always not obvious legacy of stroke sequela (mRS acuities were before the onset of 1);

(4) The degree of nerve function defect score (NIHSS) scores 4 to 24 points, body movement component (NIHSS score paragraphs 5 and 6) total score 2 points or higher;

(5) Understand and voluntarily signed informed consent.

4.3 Exclusion criteria

(1) Head imaging studies have confirmed that, encephalitis, brain tumor, brain abscess and cause similar symptoms of disease, or confirm with hemorrhagic cerebral infarction, epidural hematoma, intracranial hematoma, intraventricular hemorrhage, subarachnoid hemorrhage, etc.

(2) The serious disturbance of consciousness (Ia NIHSS score 2 points or higher);

(3) With hemorrhagic disease or have a bleeding tendency, or have a lower limb venous thrombosis;

(4) Serious abnormal liver and kidney function, liver function laboratory indexes of ALT > 3 ULN, renal laboratory ULN Cr > 1.5);

(5) A history of mental illness or dementia patients;

(6) Severe organ or other systemic disease, accompanied by any organ or system of malignant tumor, or ongoing anti-tumor treatment, the estimated lifetime < 3 months;

(7) Significant drug or alcohol abuse;

(8) Allergic constitution, as well as to two or more drugs or food allergies; This

medicine ingredients allergy or known;

(9) Have pregnancy (check blood HCG positive screening tests, namely HCG > 5 miu/mL), during the test preparation is pregnancy or lactation in women;

(10) In the past three months in other clinical trials;

(11) Researchers do not determine poor adherence, or any other suitable for patients to participate in this study.

4.4 Conditions and procedures for patients to withdraw from the study

4.4.1 Investigator-determined withdrawal

If a patient who has been enrolled in the study is not suitable to continue the study, the investigator will decide to withdraw the patient from the study.

(1) Allergic reaction or severe adverse event occurs, the study should be stopped according to the judgment of the doctor;

(2) To protect patients whose symptoms worsen after medication, they should be removed from the study and given another effective treatment;

(3) Patients developed certain comorbidities, complications, or specific physiological changes during the study that affected the assessment of efficacy and safety;

(4) The patient's compliance was poor, and the use of drugs is less than 70% or more than 120% of the prescribed amount;

(5) Patients with broken blindness of various causes during the study.

4.4.2 Subjects voluntarily withdrew from the study

According to the provisions of the informed consent, the subjects have the right to withdraw from the study halfway, or the subjects who have not explicitly proposed to withdraw from the study but no longer receive medication and testing and lose the follow-up are also "withdrawal" (or "shedding"). Efforts should be made to identify

the reasons for the subjects' withdrawal and to document them. For example, self-perception of poor efficacy, having difficulty tolerating some adverse reactions, couldn't continue to accept clinical research, economic factors, or not explain the reason for the loss of follow-up.

For patients who withdrew from the study for any reason, the researcher should keep the record and conduct a full data set analysis of adverse effects with the results of the last test carried forward as the final result.

For all patients who withdrew from the study, the researcher should fill in the study conclusion form and the withdrawal reasons in the case report form, which generally includes six types. That is, adverse events (including adverse drug reactions and allergic reactions), poor efficacy (deterioration or complications), violations of study protocols (including poor compliance), loss of follow-up (including patient self-withdrawal from the study), discontinuation by the sponsor, and others.

4.5 Case exclusion criteria

Subjects who should not be enrolled but have been enrolled, or who have completed the study but have violated some provisions of the study protocol during the study, the researcher believes that should be excluded, including:

(1) Have not taken the medicine;

(2) Without any test record after medication;

(3) Efficacy and safety evaluations are impossible due to the use of a banned drug.

(4) At the blinded review meeting, the principal investigator and statisticians decide whether the case is removed and which datasets to enter. The excluded cases should explain the reasons, and the study medical records should be kept for future reference.

5 Treatment

5.1 Dose and treatment protocol

5.1.1 Medication

(1) Experimental group

Ginkgo diterpene lactone meglumine injection of dosing of 5 ml per time, and 1 time per day. The ginkgo diterpene lactone meglumine injection (25mg) was diluted in 250 mL of sterile 0.9% sodium chloride injection.

(2) Control group

Ginkgo diterpene lactone meglumine injection simulation of dosing of 5 ml per time, and 1 time per day. The ginkgo diterpene lactone meglumine injection simulation was diluted in 250 mL of sterile 0.9% sodium chloride injection.

5.1.2 Medication precautions

(1) The pH value of this liquid is alkaline, so the infusion set made of polyvinyl chloride (PVC) must be used in clinical administration to prevent the reaction between the liquid and the infusion set, and the interaction between the liquid and the infusion set should be closely observed during medication.

(2) Before medication, patients should be carefully asked about medication history and allergy history, and patients with an allergic constitution should use it with caution.

(3) Before medication, the drug and the prepared infusion should be carefully checked. If turbidity, precipitation, discoloration, crystallization, or small rupture of the bottle are found in the infusion, they should not be used.

(4) Drug dilution should be prepared in strict accordance with the requirements of the instructions, and it is not allowed to change the type of diluent, dilution concentration, and dosage of dilution solution, and it is not allowed to use glucose

solution for dilution; After dispensing, it should be used immediately and should not be placed for a long time.

(5) TCM injection should be administered separately and not be mixed with other injections; There is no evidence of the safety and efficacy of GDLM when used in combination with other drugs.

(6) Strictly master the usage and dosage of drugs and the course of treatment. The medication should be performed according to the dosage, rates of intravenous infusion, and course of treatment recommended in the drug instructions, and should not exceed the dosage, rates of infusion, or continuous medication specified in the course of treatment. Clinical trial results showed that some adverse reactions might be related to the rapid infusion speed, so the infusion speed should be strictly controlled, which should not exceed 30 drops per minute.

(7) Medication should be performed in medical institutions where rescue conditions are available.

(8) In the process of medication, drug reaction should be closely observed, especially in the first 30 minutes of medication, if any abnormality is found, medication should be stopped immediately and active treatment measures should be taken; Patients should be observed in a medical facility for at least 30 minutes after the medication finished.

(9) If mild dizziness and headache occur after medication, the infusion speed should be reduced, and the symptoms may be alleviated or relieved.

(10) Patients with anaphylaxis or other obvious adverse reactions should stop taking the drug immediately and be treated in time.

(11) Use with caution in patients with severe heart, liver, and kidney diseases.

(12) Use with caution for frail elderly people or co-infected patients.

(13) One patient died suddenly of an acute pulmonary embolism two days

after the completion of the clinical trial, and there was no evidence that this patient's death was related to the trial medication.

(14) During medication, attention should be paid to blood pressure detection and liver function should be checked regularly.

(15) This product has not completed all reproductive toxicity tests, and the effect on offspring has not been observed. Use with caution if patients have fertility requirements.

(16) Clinical trials of this drug have not been conducted in pregnant and lactating women, children, and older adults over 70 years of age. Therefore, the efficacy and safety of this drug in pregnant and lactating women, children, and older adults over 70 years of age cannot be determined.

5.1.3 Drug incompatibility

There is no drug interaction related research on this product, therefore, mixed compatibility is strictly prohibited, and combined medication should be cautious.

5.2 Medication period

(1) Experimental group

Patients were treated continuously until 14 days after randomization.

(2) Control group

Patients were treated continuously until 14 days after randomization.

5.3 Concomitant medication

5.3.1 Concomitant medication during the study

The study period was defined as from the beginning of randomization to 90±7 days after randomization or the last follow-up.

5.3.2 Permitted concomitant medication

Treatment agents other than thrombolytic agents and neuroprotectants recommended by the Chinese Guidelines for the Diagnosis and Treatment of Acute Ischemic Stroke (2010 edition) and the Chinese Guidelines for the Secondary Prevention of Ischemic Stroke and Transient Ischemic Attack (2010 edition) were allowed during the study:

(1) General management and supportive treatment medications: Such as antipyretic drugs, dehydrating drugs, antihypertensive drugs, lipid-lowering drugs, hypoglycemic drugs, nutritional support drugs, and antibiotic therapy drugs. Mannitol is limited to reducing intracranial pressure as a dehydrating agent due to its free radical scavenging effect.

(2) Drugs to improve cerebral blood circulation:

- Antiplatelet drugs: including aspirin, clopidogrel, dipyridamole;
- Anticoagulants: including heparin, low molecular weight heparin, heparin-like, oral anticoagulants (e.g., Warfarin), and thrombin inhibitors (e.g., Agattriban);
- Defibrinase: including defibrinated, patronage, enclose, lumbrokinase, agkistrodon acutus enzyme;
- Dilatant drugs: crystal liquid (such as glucose, sodium chloride injection, etc.), blood products (such as whole blood, plasma, albumin, etc.), artificial colloidal liquid (such as gelatin, dextran, succinic acid gelatin, etc.).

Patients with underlying diseases that require long-term medication may continue to take it during the study period. If adverse reactions occur, it is up to the researcher to decide whether appropriate treatment is necessary. All drugs used simultaneously during the study should be recorded and explained in detail on the CRF form.

5.3.3 Drugs and treatments prohibited during the study

Neuroprotective drugs and thrombolytic drugs listed in the 2010 edition of the Chinese Guidelines for the Diagnosis and Treatment of Acute Ischemic Stroke were not allowed to be used in combination during the study period. Neuroprotective drugs included commercially available Edaravone, nimodipine, ganglioside, citicoline, piracetam (Naofukong), butylpheneptide, and human urokinin progenase (Ureicrin). And possibly neuroprotective drugs such as brain actin (brain protein hydrolysate), calf serum deproteinization injection, and calf blood deproteinization extract injection; Thrombolytic drugs include rt-PA and urokinase; Mannitol is also prohibited if used for neuroprotective purposes other than reducing intracranial pressure.

Drugs containing TCM components for the treatment of cerebral infarction are prohibited during the study period (including, but not limited to Breviscapine injection, Astragalus injection, ligustrazine injection, Salvia miltiorrhiza injection, Compound Salvia miltiorrhiza injection, Sodium aesculin for injection, Puerarin injection, Angelica injection, Compound Angelica injection, Honghua injection, Ginkgo damo injection, Ginkgo leaf extract injection, Acanthopujia injection, Kudiao injection, Xingnaojing injection, Xuesaitong injection/tablet/glue Sac, Xueshuantong Injection, Shuxuetong injection, Mailuoning injection, Compound Shexiang Injection, Naoxuetong oral liquid/granule, Naoxuekang Tablets/granules/capsules/oral liquid, Naoluotong tablets/capsules, Dahuoluo Wan, Xiaohuoluo Wan, Sanfeng Huoluo Wan, Huatuo Zao Wan, Xinnaoxuantong Tablet/capsule, Thrombolysing-solving capsule, Xiaoshuantongluo Tablet Etc.)

The use of unmarketed drugs or other investigational drugs is prohibited during the study. Vascular opening techniques such as mechanical thrombectomy or stents were prohibited during the study.

5.3.4 Rehabilitation training regulations

During the follow-up period, if the patient needs rehabilitation, the guidelines of Chinese Stroke Rehabilitation Treatment Guidelines 2011 should be followed, and the

researchers should inquire about the rehabilitation training of the patient in detail and record it in the study medical record and CRF.

5.4 Compliance evaluation

Compliance was evaluated by drug counting.

Medication compliance of subjects= (Dosage actually used/Dosage requirements) × 100%.

Dosage actually used = dosage delivered minus dosage returned (including remaining returned dosage and lost dosage, etc.)

Dosage requirements = The number of times the drug should be used from the last time it was issued to this visit × each prescribed dosage.

6 Efficacy evaluation

6.1 Primary efficacy measure

The proportion of mRS of 0-1 on day 90 after randomization.

✧ mRS scores range from 0 to 6, and score 0 indicates no symptoms, score 1 indicates no clinically significant disability, score 2 indicates slight disability, score 3 indicates moderate disability, score 4 indicates moderately severe disability, score 5 indicates severe disability, and score 6 indicates death.

6.2 Secondary efficacy measures

(1) The proportion of patients with mRS 0-2 on day 90;

(2) The proportion of patients with recovery of neurological deficits on day 7: decline in NIHSS score ≥4;

(3) The proportion of patients with recovery of neurological deficits on day 14: decline in NIHSS score ≥4;

(4) The proportion of patients with deterioration of neurological deficits on day 7:

increase in NIHSS score increase in NIHSS score ≥4;

(5) The proportion of patients with deterioration of neurological deficits on day 7: increase in NIHSS score increase in NIHSS score ≥3;

(6) The proportion of patients with deterioration of neurological deficits on day 7: increase in NIHSS score increase in NIHSS score ≥2;

(7) The proportion of patients with deterioration of neurological deficits on day 7: increase in NIHSS score increase in NIHSS score ≥1.

6.3 Exploratory measures

(1) MoCA from baseline to day 14;

(2) MoCA from baseline to day 90;

(3) Quality of Life (EuroQol EQ-5D scale) from baseline to day 14;

✧ The EQ-5D consists of five dimensions (mobility, self-care, usual activities, pain or discomfort, and anxiety or depression) and a subjectively rated visual analog scale. EQ-5D utility-scoring algorithms will be used from three conversion tables from the United Kingdom, the United States, and Japan, based on time trade-off models.

(4) Quality of Life (EuroQol EQ-5D scale) from baseline to day 90;

(5) The rate of all-cause mortality within 90 days;

(6) The rate of recurrent ischemic stroke within 90 days;

(7) The rate of combined vascular events within 90 days, including stroke, myocardial infarction, vascular death, and angina pectoris.

7 Safety evaluation

7.1 Safety measures

(1) Adverse events within 90 days;

(2) Severe adverse events within 90 days;

(3) Vital signs and laboratory variables from baseline to day 14 after randomization, including blood pressure, heart rate, breathing, body temperature, blood routine (hemoglobin, platelet count, red blood cell, white blood cell count), urine routine (red blood cell count, white blood cell count, glucose, protein), and blood biochemistry (alkaline phosphatase, alanine aminotransferase, aspartate transaminase, γ-glutamyl transpeptidase, total bilirubin, urea nitrogen, creatinine)

7.2 Adverse events

7.2.1 Definition of adverse events

Any adverse medical event occurring from the start of patient randomization to the last follow-up visit, regardless of whether it was causally related to the trial drug, was judged to be an adverse event. Any event that occurs in a subject in a clinical study that is not expected to occur is an adverse event. The study physician should report in concise medical terms all adverse events directly observed by the physician or spontaneously reported by the subject patient. Adverse drug reactions are those that are directly and indirectly related to the drug. If there is difficulty in determining the relationship to the drug, to prevent omission, any adverse event that occurs should be recorded on a case record form, documenting the time of occurrence, severity, duration, measures taken, and regression of the adverse event, and its possible correlation with the drug, respectively.

7.2.2 Observation and recording of adverse events

Safety evaluation of all subjects receiving at least 1 dose will be performed and all the following events will be recorded: adverse events, severe adverse events, death, stroke recurrence, discontinuation for any reason, abnormal laboratory tests, changes

in vital signs, and changes in physical examination findings. Adverse events are recorded from the start of randomization to 90 days of enrollment or during the last follow-up visit. If an adverse event occurs, it will be followed until the adverse event disappears or returns to baseline levels or is not clinically significant.

All adverse events that occur during the study will be recorded on a case report form. The assessment of adverse events will include the name of the adverse event, severity, regression, relationship to the trial drug, and management measures.

7.2.3 Severity of adverse events

Classify the severity of adverse events into three levels: mild, moderate, and severe, defined as follows.

1) Mild - minor symptoms or disease that improves quickly after discontinuation and does not require treatment;
2) Moderate-causing transient damage, not requiring hospitalization or extended hospitalization, requiring treatment or intervention, easily recovered;
3) Severe-causes transient damage requires hospitalization for outpatients, extended hospitalization for inpatients (more than 7 days), causes permanent system/organ damage or is life threatening (e.g., symptoms requiring emergency care such as asphyxia, shock, coma, etc.).

7.2.4 Correlation of adverse events with drugs

The investigator should assess the possible association between the adverse event and the study drug and the combination drug, as assessed by the following 5-level classification.

1) Definitely related: the reaction appears in a reasonable chronological order after administration and the reaction is consistent with the type of reaction known for the suspected drug; improves after discontinuation and the reaction reappears with repeated administration and cannot be explained by the subject's disease or coadministration;

2) Likely related: Reaction appears in a reasonable chronological order after dosing and the reaction is consistent with the type of reaction known for the suspected drug; improves after discontinuation and cannot be explained by the subject's disease or coadministration;
3) Possibly related: the reaction appears in a reasonable time sequence after administration and the reaction is consistent with the type of reaction known for the suspected drug; the patient's clinical status or other treatment modality may also have produced the reaction;
4) Possibly unrelated: The reaction does not appear in a reasonable time sequence after administration, and the reaction does not correspond to the known reaction type of the suspected drug; the patient's clinical status or other treatment modality may produce the reaction;
5) Unrelated: the reaction does not occur in a reasonable time sequence after administration, the reaction is consistent with the type of reaction known for the non-test drug; the patient's clinical status or other treatment modality may produce the reaction, the disease status improves or the reaction is eliminated by stopping other treatment modalities, and the reaction occurs by repeating other treatment modalities.

7.3 Severe adverse events

A severe adverse event is defined as an adverse event that results in

1) Resulting in death;
2) Life-threatening (means that the subject is at risk of death at the time of an adverse event. It does not refer to those adverse events that could lead to death assuming a more aggravated condition);
3) requiring hospitalization or extended hospitalization;
4) Resulting in persistent or severe disability or insufficiency;
5) Result in a congenital anomaly or birth defect;

6) Medical events that, in the opinion of the investigator, can be judged as severe adverse events.

If a severe adverse event occurs in a subject during the trial, whether it is related to the trial drug, the investigator should immediately take appropriate therapeutic measures for the subject to safeguarding the safety of the subject, and the investigator must report to the supervisor, the ethics committee of the Center and the undertaking unit within 24 hours. The undertaking unit reports to the ethics committee, the sponsor, and simultaneously to the State Food and Drug Administration for the record. The investigator must complete the severe adverse event form.

Any severe adverse event that is not resolved at the end of the trial or at the time of the subject's early withdrawal must be followed until one of the following is reached:

1) The event disappears or resolves;
2) Events are stable;
3) Events return to baseline levels (if baseline values are available);
4) Remission of events to no clinical significance;
5) The event can be attributed to a drug other than the investigational drug or factors unrelated to the conduct of the study, or when more information is unlikely to be available (patient or healthcare provider refuses to provide more information, or there is evidence patients are lost to follow-up despite best efforts).

7.4 Recording, processing, and reporting

- ✧ Any adverse events occurring during the trial, whether related to the trial drug or not, should be recorded;
- ✧ Adverse events related to the trial drug must be handled carefully;
- ✧ Subjects should be asked to truthfully report the changes in their condition after taking the drug;

- ✧ The researcher should avoid leading questions. While observing the curative effect, pay close attention to the adverse events or unanticipated side effects (including symptoms, signs, and laboratory tests), analyze the reasons, make judgments, and follow up observations and records;
- ✧ The incidence of adverse events should be counted;
- ✧ For adverse events occurring during the trial, the symptoms, degree, time of appearance, duration, treatment measures, outcome, relationship with drugs, and follow-up methods should be recorded in detail in the case report form, signed and dated;
- ✧ When an adverse event is found, the researcher can decide whether to suspend the study according to the condition, conduct a follow-up investigation on the cases of drug discontinuation due to the adverse event, and record the treatment process and results in detail;
- ✧ Once a severe adverse event or important adverse event occurs in a clinical trial, the investigator must initiate emergency procedures;
- ✧ All subjects who use the therapeutic drug, regardless of whether they complete the whole process of the trial or not, those who have adverse events should be included in the adverse event statistics.

7.5 Emergency Procedures

Once a severe adverse event or important adverse event occurs in a clinical trial, the investigator must immediately organize the rescue of the subjects to ensure the safety of the subjects. Regardless of whether it is related to the study drug, and whether the study drug has been given, the responsible unit must be notified immediately by telephone/fax. Detailed reporting procedures can be found in the research center folder. Subsequently, the clinical monitor must provide a written report describing the circumstances and results of the event. In addition to confirming the contents of the telephone/fax report, the written report must also report other failures. The clinical monitor should check the written report and report it to the drug

safety department within 24 hours after receiving the telephone report or no later than the 2nd working day according to the GCP standard operating procedures.

Contact person for severe adverse event reporting

Unit Name	phone number	Fax
Beijing Tiantan Hospital, Capital Medical University	010-67098555	/
Jiangsu Kangyuan Pharmaceutical Co., Ltd.	0518-85521999	0518-85521983
Beijing Bonovo Pharmaceutical Technology Development Co., Ltd.	010-65773066	010-65791599
Department of Safety Supervision, State Food, and Drug Administration	010-68313344-1013	010-68313182

8 Data management and statistical analysis

8.1 Independent Data and Safety Monitoring Board (DSMB)

An independent DSMB is established to review safety and disability data in a non-blind setting, which is independent of the researchers, CRO, and investigators who specifically assessed the subject. The independent DSMB can modify or discontinue studies based on unforeseen safety issues or unexplained increased disability or progress.

8.2 Data management

The investigator should complete the Case Report Form (CRF) based on the individuals' original observation records timely, completely, accurately, and clearly, following the CRF completion guidelines.

During the conduction of the study, the auditor has the responsibility to monitor whether the research is being conducted following the protocol. This involves verifying that all CRFs are completed correctly and consistent with the original data. In case there are errors or omissions, the auditor should promptly ask the investigator to correct them. To ensure proper documentation, the original record should be kept visible when revising, and the correction should be signed and dated by the investigator.

Once the monitor has verified the accuracy, the CRF should be signed by both the investigator and the monitor, and promptly sent to the data manager. The CRF should be sent, transferred, and received following the standard operating procedures, and should be signed by the investigator, auditor, and data manager.

For queries that exist in the case report form, the data manager will generate a data rating questionnaire (DRQ) and send a query to the investigator through the clinical monitor, who should answer and return it as soon as possible, and the data manager will correct and confirm the data according to the investigator's answer, and if necessary, the DRQ can be sent again. Coding standards, processes, and dictionaries are established and ensured to be correct, safe, and confidential.

Double entry is adopted during the input process. If any problem or accident is found in the input process, it shall be registered and reported in time, to solve the problem quickly. Additionally, after data entry is complete, it is recommended to randomly check a portion of the observation grids to assess input quality, identify any existing problems, and take corrective measures as needed.

Data managers, supervisors, medical staff, and statisticians perform data verification following the Data Validation Plan (DVP), data verification content, methods, and verification requirements.

The original CRF should be archived and stored in numbered order with a retrieval catalog for reference. Electronic data files, including databases, check programs, analysis programs, analysis results, coding books, and description files, should be stored in categories with multiple backups on different disks or recording media to prevent damage. All original records should be kept following the "Quality Control Standards for Drug Clinical Trials".

8.3 Statistical analysis

8.3.1 Sample Size Estimate

The study hypothesized that there is no difference in the rate of mRS of 0-1 on day 90. According to the results of previous studies, it is assumed that the proportion of patients in the placebo group with mRS of 0-1 is 45%, the proportion of patients in the ginkgo diterpenelactone meglumine injection group with mRS of 0-1 is 50%. When the test level is 0.05, the power is 0.80, 1,565 patients are needed in each group. Considering an approximate 10% loss of follow-up, a total of 3,452 patients are needed in this study, with 1,726 patients in each group. PASS11 (NCSS, LLC) will be used to calculate the sample size.

8.3.2 General Principles

Biostatisticians and primary investigators formulate statistical analysis plans according to the research plan, and finalize the protocol before data locking. All programming will be performed using SAS Version 9.4.

For continuous variables, mean, median, standard deviation, maximum, minimum, 25th, and 75th of the subjects that are not missing will be listed. For categorized or ordinal variables, frequency tables (frequencies and percentages) of the subjects that are not missing will be listed.

8.3.2.1 Test Level

All hypotheses will be tested by a two-sided type I error of 0.05. All statistics were two-sided with a $P<0.05$ considered significant. The reliability of all confidence intervals (CIs) will be 95%.

8.3.2.2 Hypothesis Test

The primary outcome of this study is the proportion of mRS 0-1 on day 90 after randomization. In this study, the null hypothesis of no difference in the rate of mRS

0-1 on day 90 between the two treatment groups will be tested using a two-sided test at the 5% level of significance.

H0: $\lambda_1=\lambda_2$

H1: $\lambda_1\neq\lambda_2$

Where λ_1 is the rate of mRS 0-1 on day 90 in the ginkgo diterpenelactone meglumine injection group and λ_2 is the rate in the placebo group.

8.3.2.3 Missing Data

Missing data on the primary efficacy outcome of mRS score will be filled in using the last observation carry-forward (LOCF) estimation method. Missing data in the secondary efficacy outcomes will not be filled.

8.3.3 Study Population

8.3.3.1 Disposition of Patients

The number of subjects in each analysis population will be presented. Subjects to be excluded from the Per Protocol Set (PPS) population will be listed, and the total number of subjects attending each clinic visit will also be summarized. The number of subjects randomized, completed, and prematurely withdrawn from the study will be presented for each treatment group. The primary reasons for withdrawal both prior to and post randomization will also be presented. A data display listing and summary of deviations from the inclusion/exclusion criteria will be presented for all subjects who were either entered or randomized into the trial.

8.3.3.2 Protocol Violations and Deviations

Subject data will be examined for evidence of protocol violators to assess how well the protocol was followed. Inclusion and exclusion criteria are detailed in section 4.3 and 4.4.

Subjects who commit protocol violations will be included in the Full Analysis Set (FAS) Population but not in the PPS Population. These protocol violations will be shown in a listing. Subjects can either be full or partial protocol violators. A full protocol violator is completely excluded from the PPS Population. A partial protocol violator has only some data excluded. For subjects who violated the protocol during the treatment period due to unpermitted changes in the treatment or prohibited concurrent treatment, the analysis will only use data recorded prior to the violation. For all violations which reference the treatment period, the treatment start date will be used as the reference date.

A listing of all possible protocol violators will be produced for clinical review. The final list of subjects who are protocol violators and are therefore excluded from the Per-Protocol population will be agreed by the study team.

8.3.4 Demographic and Baseline Characteristics

Demographic characteristics, including age, sex, ethnic, and body mass index, will be listed and summarized for subjects in each treatment group. This will also be done for medical history (including stroke, hypertension, and diabetes), and NIHSS score.

Vital signs, including systolic blood pressure, and diastolic blood pressure, will also be listed and summarized in each treatment group.

The continuous data under normal distribution will be presented as mean and standard deviation and the continuous data under skewness distribution will be presented as median and interquartile range; categorical data will be presented as n (%). T-test or Wilcoxon rank sum test will be used for comparison between two continuous data, and Chi-squared tests or Fisher exact test will be used for comparison between two categorical data.

8.3.5 Efficacy Analyses

8.3.5.1 Primary Efficacy Analysis

The primary outcome is the proportion of patients with mRS of 0-1on day 90 after randomization. FAS will be the primary population for efficacy analyses. PPS will be used as the secondary population for the efficacy analyses. If the results in the PPS population are inconsistent with the FAS population, a detailed analysis of the inconsistent results is required.

The differences in the proportions of the primary outcome (mRS 0-1 on day 90) between treatment groups, and their corresponding 95% CIs will be estimated based on Newcombe-Wilson. The odds ratios (ORs) and 95% CIs of the primary outcome will be estimated with the logistic regression.

Interactions with Subgroups

Summary tables will be produced for the predefined subgroups. Interactions between treatment and these subgroups will be investigated, using a logistic regression model. A separate model will be used for each interaction to determine its significance. This will also be presented graphically on a forest plot.

The predefined subgroups include:

- Age (<65/≥65)
- Sex (male/female)
- Previous stroke (yes/no)
- Hypertension (yes/no)
- Diabetes mellitus (yes/no)
- Time from onset to treatment (<24/≥24)
- NIHSS score (≤7/>7)

8.3.5.2 Secondary Efficacy Analyses

(1) The proportion of patients with mRS 0-2 on day 90

The differences in the proportions of patients with mRS 0-2 on day 90 between treatment groups, and their corresponding 95% CIs, will be estimated based on Newcombe-Wilson. The ORs and 95% CIs will be estimated with the logistic regression.

(2) The proportion of patients with recovery of neurological deficits on day 7: decline in NIHSS score ≥4

The differences in the proportions of patients with recovery of neurological deficits between treatment groups, and their corresponding 95% CIs, will be estimated based on Newcombe-Wilson. The ORs and 95% CIs will be estimated with the logistic regression.

(3) The proportion of patients with recovery of neurological deficits on day 14: decline in NIHSS score ≥4

The differences in the proportions of patients with recovery of neurological deficits between treatment groups, and their corresponding 95% CIs, will be estimated based on Newcombe-Wilson. The ORs and 95% CIs will be estimated with the logistic regression.

(4) The proportion of patients with deterioration of neurological deficits on day 7: increase in NIHSS score ≥4

The differences in the proportions of patients with deterioration of neurological deficits between treatment groups, and their corresponding 95% CIs, will be estimated based on Newcombe-Wilson. The ORs and 95% CIs will be estimated with the logistic regression.

(5) The proportion of patients with deterioration of neurological deficits on day 7: increase in NIHSS score ≥3

The differences in the proportions of patients with deterioration of neurological deficits between treatment groups, and their corresponding 95% CIs, will be estimated based on Newcombe-Wilson. The ORs and 95% CIs will be estimated with the logistic regression.

(6) The proportion of patients with deterioration of neurological deficits on day 7: increase in NIHSS score increase in NIHSS score ≥2

The differences in the proportions of patients with deterioration of neurological deficits between treatment groups, and their corresponding 95% CIs, will be estimated based on Newcombe-Wilson. The ORs and 95% CIs will be estimated with the logistic regression.

(7) The proportion of patients with deterioration of neurological deficits on day 7: increase in NIHSS score increase in NIHSS score ≥1

The differences in the proportions of patients with deterioration of neurological deficits between treatment groups, and their corresponding 95% CIs, will be estimated based on Newcombe-Wilson. The ORs and 95% CIs will be estimated with the logistic regression.

8.3.5.3 Exploratory Efficacy Analyses

(1) MoCA change from baseline to day 14

Differences between treatment groups in MoCA will be calculated as mean differences with 95% CIs by using a generalized linear model if the data are normal, and the t-test will be used for comparison between groups. If the data are skewed, the median differences and 95% CIs will be calculated by using a Hodges-Lehmann estimation, and a 2-sample Wilcoxon rank-sum test will be used for comparison between treatment groups.

(2) MoCA change from baseline to day 90

Differences between treatment groups in MoCA will be calculated as mean differences with 95% CIs by using a generalized linear model if the data are normal, and the t-test will be used for comparison between groups. If the data are skewed, the median differences and 95% CIs will be calculated by using a Hodges-Lehmann estimation, and a 2-sample Wilcoxon rank-sum test will be used for comparison between treatment groups.

(3) Quality of Life (EuroQol EQ-5D scale) change from baseline to day 14

Differences between treatment groups in the EQ-5D scale will be calculated as mean differences with 95% CIs by using a generalized linear model if the data are normal, and the t-test will be used for comparison between groups. If the data are skewed, the median differences and 95% CIs will be calculated by using a Hodges-Lehmann estimation, and a 2-sample Wilcoxon rank-sum test will be used for comparison between treatment groups.

(4) Quality of Life (EuroQol EQ-5D scale) change from baseline to day 90

Differences between treatment groups in the EQ-5D scale will be calculated as mean differences with 95% CIs by using a generalized linear model if the data are normal, and the t-test will be used for comparison between groups. If the data are skewed, the median differences and 95% CIs will be calculated by using a Hodges-Lehmann estimation, and a 2-sample Wilcoxon rank-sum test will be used for comparison between treatment groups.

(5) The rate of all-cause mortality within 90 days

The rate of all-cause mortality within 90 days will be analyzed using a Cox proportional hazard model. The hazard ratio and 95% CI will be reported.

(6) The rate of recurrent ischemic stroke within 90 days

The rate of recurrent ischemic stroke within 90 days will be analyzed using a Cox proportional hazard model. The hazard ratio and 95% CI will be reported.

(7) The rate of combined vascular events within 90 days, including stroke, myocardial infarction, vascular death, and angina pectoris

The rate of combined vascular events within 90 days will be analyzed using a Cox proportional hazard model. The hazard ratio and 95% CI will be reported.

8.3.6 Safety Analysis

All analyses of safety data will be carried out using the safety set (SS) population.

8.3.6.1 Extent of Exposure and Compliance

Study duration (days) = (end date of study - start date of study) + 1

Duration of treatment (days) = (end date of study treatment - start date of study treatment) + 1

Compliance = actual dose / planned dosage × 100%. A good compliance is defined as the compliance between 70% and 120%.

8.3.6.2 Adverse events (AE)

Adverse event data will be coded according to the current version of MedDRA. Treatment Emergent Adverse Event (TEAE) will be used in the analysis, while adverse events that start prior to the first dose of study medication (pre-treatment) will not be involved into the safety analysis. Adverse events on study medication (during treatment) and after the last dose of study medication (post-treatment) will be analyzed.

The differences in the proportions of patients with AE between treatment groups and their corresponding 95% CIs will be estimated based on Newcombe-Wilson. The ORs and 95% CIs will be estimated with the logistic regression. Chi-squared test or Fisher’s Exact test will be used to compare the number of each AE combination according to system organ class and preferred term between treatment groups.

8.3.6.3 Severe Adverse events (SAE)

The differences in the proportions of patients with SAE between treatment groups and their corresponding 95% CIs will be estimated based on Newcombe-Wilson. The ORs and 95% CIs will be estimated with the logistic regression. Chi-squared test or Fisher’s Exact test will be used to compare the number of each SAE combination according to system organ class and preferred term between treatment groups.

8.3.6.4 Vital Signs and Laboratory Test Evaluation

Within each treatment group, the number and percentage of subjects with elevated in vital signs from baseline to day 14 after randomization will be summarized. Chi-squared test or Fisher’s Exact test will be used to compare the difference between treatment groups. The baseline measurements and changes after treatment of blood routine and blood biochemistry will be evaluated by the researchers and described as "abnormal and clinically significant" when appropriate.

Within each treatment group, the number and percentage of subjects with an "abnormal and clinically significant" value will be summarized and Chi-squared test or Fisher’s Exact test will be used to compare the difference between treatment groups.

9 Quality control and quality assurance

9.1 Quality control

(1) Researchers' qualifications

Researchers participating in the clinical trial must be qualified and have the professional background and ability about clinical study.

(2) Researchers' training

Before the start of the trial and after approval by the institutional review boards (IRB), the CRA will go to each center and convene all participating investigators to participate in start-up training, including the protocol, the inclusion and exclusion criteria, the treatment of AEs, and the completion of the CRF and SAE forms.

(3) Clinical trial monitoring

Clinical trial monitors assigned by Beijing Bionowo Pharmaceutical Technology Development Co., LTD. will conduct regular inspection and visit on the conduction and completion of the study. The monitors will check the completeness of case records, the accuracy of CRFs, the study data, and the compliance with study protocols and good clinical practice (GCP). They will also investigate the progress of enrolled patients, and ensure the accuracy of study drug storage, dispensing, and counts. Investigators and study staff should assist the job of clinical trial monitors.

9.2 Quality assurance

The Quality Assurance Department of Beijing Bionowo Pharmaceutical Technology Development Co., LTD. is responsible for investigating and reviewing the conduction of the clinical trial for compliance with the company's internal standard operating procedures (SOPs) and assessing compliance with GCP and relevant regulations.

10 Ethical Principles

Prior to the conduction of this trial, the study protocol, the prepared informed consent form, and other information given to patients must be submitted to the Institutional Review Board (IRB) for review. A written approval by the IRB must be obtained before the start of the study. Any amendments to the protocol expect for the administrative amendments must be approved by this committee.

The trial can be conducted only after informed consent was obtained from participants. The rights and interests of the participants should be ensured during the study. Data of participants should be kept confidential.

11 Data preservation and conclusion

The investigators must keep the original data of each participant (usually in the medical records), and any information appearing on the CRF should be traceable to the original data. The original data includes an informed consent form signed by the patient with the study number and study name, laboratory data, electrocardiograms, etc.

The basic data of the participants should be kept for a sufficient period (usually 5 years after the end of the trial) until the Beijing Bionowo Pharmaceutical Technology Development Co., LTD. notify the investigators/institutions that the trial records will no longer be kept.

Basic data includes:

- ✧ IRB's approval document of the study protocol and its revisions
- ✧ All original materials
- ✧ CRF
- ✧ Informed Consent Form
- ✧ Any other study-related documents

12 Responsibilities assumed by all parties

The researchers should keep all information strictly confidential. Other study staff and IRB are also required to adopt the same confidentiality measures. The experimental drug, control drug, and simulation for this study were provided by Jiangsu Kanion Pharmaceutical Co., LTD. Beijing Bionowo Pharmaceutical Technology Development Co., LTD. was responsible for monitoring the process of the clinical trial.

13 References

[1] State Food and Drug Administration. Good Clinical Practice (2003).

[2] Neurology CSo, Society CS. Chinese guidelines for diagnosis and treatment of acute ischemic stroke 2010. Chinese Journal of Neurology. 2010;(14):16-19. doi:10.3760/cma.j.issn.1006-7876.2010.02.022

[3] Neurology CSo, Society CS. Chinese guideline for the secondary prevention of ischemic stroke and transient ischemic attack 2010. Chinese Journal of Neurology. 2010;(02):154-160.

[4] Pharmacodynamics and phase I, II, and III clinical trial data of Ginkgo biloba diterpene lactone glucosamine injection.

[5] Gao D, Zhang L. Guidelines for monitoring adverse drug reactions. China Medical Science and Technology Press; 1996.

[6] Wang S. Points to consider on clinical trials of medicinal products for the treatment of acute ischemic stroke. The Chinese Journal of Clinical Pharmacology. 2010;26(08):563-567.

[7] Sacco RL, Kasner SE, Broderick JP, et al. An updated definition of stroke for the 21st century: a statement for healthcare professionals from the American Heart Association/American Stroke Association. Stroke. 2013;44(7):2064-2089. doi:10.1161/STR.0b013e318296aeca

[8] Liu X, Xia J, Wang L, et al. Efficacy and safety of ginsenoside-Rd for acute ischaemic stroke: a randomized, double-blind, placebo-controlled, phase II multicenter trial. Eur J Neurol. 2009;16(5):569-575. doi:10.1111/j.1468-1331.2009.02534.x

[9] Liu X, Wang L, Wen A, et al. Ginsenoside-Rd improves outcome of acute ischaemic stroke - a randomized, double-blind, placebo-controlled, multicenter trial. Eur J Neurol. 2012;19(6):855-863. doi:10.1111/j.1468-1331.2011.03634.x

[10] Siegler JE, Boehme AK, Kumar AD, et al. Identification of modifiable and nonmodifiable risk factors for neurologic deterioration after acute ischemic stroke. J Stroke Cerebrovasc Dis. 2013;22(7):e207-e213. doi:10.1016/j.jstrokecerebrovasdis.2012.11.006

[11] Miyamoto N, Tanaka Y, Ueno Y, et al. Demographic, clinical, and radiologic predictors of neurologic deterioration in patients with acute ischemic stroke. J Stroke Cerebrovasc Dis. 2013;22(3):205-210. doi:10.1016/j.jstrokecerebrovasdis.2011.07.018

[12] Shuaib A, Lees KR, Lyden P, et al. NXY-059 for the treatment of acute ischemic stroke. N Engl J Med. 2007;357(6):562-571.

[13] Yang K. The Clinical Research of Pathogenesis Mechanisms and Risk Factors of Deterioration of Neurological Function in Acute Cerebral Infarction. 硕士. Kunming Medical College; 2009.

[14] Fan S, Wang T, He B, Wei D, Liu J, Ma P. Study on related factors of deteriorating cerebral infarction. Chinese Journal of Critical Care Medicine. 2008;28(12):1118-1121.

[15] Feng Y, Zheng Z. Analysis of the etiology, risk factors and clinical characteristics of progressive cerebral infarction. Chinese Journal of Practical Nervous Diseases. 2009;12(04):39-40.

Annex 1: National Institutes of Health Stroke Scale (NIHSS)

Administer stroke scale items in the order listed. Record performance in each category after each subscale exam. Do not go back and change scores. Follow directions provided for each exam technique. Scores should reflect what the patient does, not what the clinician thinks the patient can do. The clinician should record answers while administering the exam and work quickly. Except where indicated, the patient should not be coached (i.e., repeated requests to patient to make a special effort).

If some items are not assessed, they should be detailed in the form.

✧ Handedness: 1-Left 2-Right 99-Uncertain

	Instructions	Scale Definition	Score
**1a**	**Level of consciousness**: The investigator must choose a response if a full evaluation is prevented by such obstacles as an endotracheal tube, language barrier, orotracheal trauma/bandages. A 3 is scored only if the patient makes no movement (other than reflexive posturing) in response to noxious stimulation	0 = Alert; keenly responsive.<br>1 = Not alert; but arousable by minor stimulation to obey, answer, or respond.<br>2 = Not alert; requires repeated stimulation to attend or is obtunded and requires strong or painful stimulation to make movements (not stereotyped).<br>3 = Responds only with reflex motor or autonomic effects or totally unresponsive, flaccid, and areflexic.	____
**1b**	**LOC Questions**: The patient is asked the month and his/her age. The answer must be correct - there is no partial credit for being close. Aphasic	0 = Answers both questions correctly.<br>1 = Answers one question correctly.	____

	**Instructions**	**Scale Definition**	**Score**
	and stuporous patients who do not comprehend the questions will score 2. Patients unable to speak because of endotracheal intubation, orotracheal trauma, severe dysarthria from any cause, language barrier, or any other problem not secondary to aphasia are given a 1. It is important that only the initial answer be graded and that the examiner not "help" the patient with verbal or non-verbal cues.	2 = Answers neither question correctly.	
1c	**LOC Commands**: The patient is asked to open and close the eyes and then to grip and release the non-paretic hand. Substitute another one step command if the hands cannot be used. Credit is given if an unequivocal attempt is made but not completed due to weakness. If the patient does not respond to command, the task should be demonstrated to him or her (pantomime), and the result scored (i.e., follows none, one or two commands). Patients with trauma, amputation, or other physical impediments should be given suitable one-step commands. Only the first attempt is scored.	0 = Performs both tasks correctly.<br>1 = Performs one task correctly.<br>2 = Performs neither task correctly.	____

	Instructions	Scale Definition	Score
**2**	**Best Gaze**: Only horizontal eye movements will be tested. Voluntary or reflexive (oculocephalic) eye movements will be scored, but caloric testing is not done. If the patient has a conjugate deviation of the eyes that can be overcome by voluntary or reflexive activity, the score will be 1. If a patient has an isolated peripheral nerve paresis (CN III, IV or VI), score a 1. Gaze is testable in all aphasic patients. Patients with ocular trauma, bandages, pre-existing blindness, or other disorder of visual acuity or fields should be tested with reflexive movements, and a choice made by the investigator. Establishing eye contact and then moving about the patient from side to side will occasionally clarify the presence of a partial gaze palsy.	0 = Normal.<br>1 = Partial gaze palsy; gaze is abnormal in one or both eyes, but forced deviation or total gaze paresis is not present.<br>2 = Forced deviation, or total gaze paresis not overcome by the oculocephalic maneuver.	____
**3**	**Visual**: Visual fields (upper and lower quadrants) are tested by confrontation, using finger counting or visual threat, as appropriate. Patients may be encouraged, but if they look at the side of the moving fingers appropriately, this can be	0 = No visual loss.<br>1 = Partial hemianopia.<br>2 = Complete hemianopia.<br>3 = Bilateral hemianopia (blind including cortical blindness).	____

	Instructions	Scale Definition	Score
	scored as normal. If there is unilateral blindness or enucleation, visual fields in the remaining eye are scored.<br>Score 1 only if a clear-cut asymmetry, including quadrantanopia, is found. If patient is blind from any cause, score 3. Double simultaneous stimulation is performed at this point. If there is extinction, patient receives a 1, and the results are used to respond to item 11.		
**4**	**Facial Palsy**: Ask – or use pantomime to encourage – the patient to show teeth or raise eyebrows and close eyes. Score symmetry of grimace in response to noxious stimuli in the poorly responsive or non-comprehending patient. If facial trauma/bandages, orotracheal tube, tape or other physical barriers obscure the face, these should be removed to the extent possible.	0 = Normal symmetrical movements.<br>1 = Minor paralysis (flattened nasolabial fold, asymmetry on smiling).<br>2 = Partial paralysis (total or near-total paralysis of lower face).<br>3 = Complete paralysis of one or both<br>sides (absence of facial movement in the upper and lower face).	____
**5**	**Motor Arm**: The limb is placed in the appropriate position: extend the arms (palms down) 90 degrees (if sitting) or 45 degrees (if supine).	0 = No drift; limb holds 90 (or 45) degrees for full 10 seconds.<br>1 = Drift; limb holds 90 (or 45) degrees, but drifts down before	Left: ____<br>Right: ____

	Instructions	Scale Definition	Score
	Drift is scored if the arm falls before 10 seconds. The aphasic patient is encouraged using urgency in the voice and pantomime, but not noxious stimulation. Only the affected side is tested.	full<br>10 seconds; does not hit bed or other support.<br>2 = Some effort against gravity; limb cannot get to or maintain (if cued) 90 (or 45) degrees, drifts down to bed, but has some effort against gravity.<br>3 = No effort against gravity; limb falls.<br>4 = No movement.<br>9 = Amputation or joint fusion, explain:	
**6**	**Leg**: The limb is placed in the appropriate position: hold the leg at 30 degrees (always tested supine). Drift is scored if the leg falls before 5 seconds. The aphasic patient is encouraged using urgency in the voice and pantomime, but not noxious stimulation. Only the affected side is tested.	0 = No drift; leg holds 30-degree position for full 5 second<br>1 = Drift; leg falls by the end of the 5-second period but does not hit bed.<br>2 = Some effort against gravity; leg<br>falls to bed by 5 seconds but has some<br>effort against gravity.<br>3 = No effort against gravity; leg falls to bed immediately.<br>4 = No movement.<br>9 = Amputation or joint fusion,	Left:<br>____<br>Right:<br>____

	Instructions	Scale Definition	Score
		explain:	
**7**	**Limb Ataxia**: This item is aimed at finding evidence of a bilateral cerebellar lesion. Test with eyes open. In case of visual defect, ensure testing is done in intact visual field. The finger-nose-finger and heel-shin tests are performed on both sides, and ataxia is scored only if present out of proportion to weakness. Ataxia is absent in the patient who cannot understand or is paralyzed. Only in the case of amputation or joint fusion, the examiner should record the score as 9, and clearly write the explanation for this choice. In case of blindness, test by having the patient touch nose from extended arm position.	0 = Absent.<br>1 = Present in one limb.<br>2 = Present in two limbs.<br>9 = Amputation or joint fusion, explain: __________	____
**8**	**Sensory**: Sensation or grimace to pinprick when tested, or withdrawal from noxious stimulus in the obtunded or aphasic patient. Only sensory loss attributed to stroke is scored as abnormal and the examiner should test as many body areas (arms [not hands], legs, trunk, face) as needed to accurately check for hemisensory loss. A score of 2,	0 = Normal; no sensory loss.<br>1 = Mild-to-moderate sensory loss; patient feels pinprick is less sharp or is dull on the affected side; or there is a loss of superficial pain with pinprick, but patient is aware of being touched.<br>2 = Severe to total sensory loss; patient is not aware of being	____

	Instructions	Scale Definition	Score
	"severe or total sensory loss," should only be given when a severe or total loss of sensation can be clearly demonstrated. Stuporous and aphasic patients will, therefore, probably score 1 or 0. The patient with brainstem stroke who has bilateral loss of sensation is scored 2. If the patient does not respond and is quadriplegic, score 2. Patients in a coma (item 1a=3) are automatically given a 2 on this item.	touched in the face, arm, and leg.	
**9**	**Best Language**: A great deal of information about comprehension will be obtained during the preceding sections of the examination. For this scale item, the patient is asked to describe what is happening in the attached picture, to name the items on the attached naming sheet and to read from the attached list of sentences. Comprehension is judged from responses here, as well as to all of the commands in the preceding general neurological exam. If visual loss interferes with the tests, ask the patient to identify objects placed in the hand, repeat, and produce speech.	0 = No aphasia; normal.<br>1 = Mild-to-moderate aphasia; some obvious loss of fluency or facility of comprehension, without significant limitation on ideas expressed or form of expression. Reduction of speech and/or comprehension, however, makes conversation about provided materials difficult or impossible. For example, in conversation about provided materials, examiner can identify picture or naming card content from patient's response.	_____

	Instructions	Scale Definition	Score
	The intubated patient should be asked to write. The patient in a coma (item 1a=3) will automatically score 3 on this item. The examiner must choose a score for the patient with stupor or limited cooperation, but a score of 3 should be used only if the patient is mute and follows no one-step commands.	2 = Severe aphasia; all communication is through fragmentary expression; great need for inference, questioning, and guessing by the listener. Range of information that can be exchanged is limited; listener carries burden of communication. Examiner cannot identify materials provided from patient response.<br>3 = Mute, global aphasia; no usable speech or auditory comprehension.	
**10**	**Dysarthria**: If patient is thought to be normal, an adequate sample of speech must be obtained by asking patient to read or repeat words from the attached list. If the patient has severe aphasia, the clarity of articulation of spontaneous speech can be rated. Only if the patient is intubated or has other physical barriers to producing speech, the examiner should record the score as 9, and clearly write an explanation for this choice. Do not tell the patient why he or she is being tested.	0 = Normal.<br>1 = Mild-to-moderate dysarthria; patient slurs at least some words and, at worst, can be understood with some difficulty.<br>2 = Severe dysarthria; patient's speech is so slurred as to be unintelligible in the absence of or out of proportion to any dysphasia, or is mute/anarthric.<br>9 = Intubated or other physical barrier, explain:____________	____

	Instructions	Scale Definition	Score
**11**	**Extinction and Inattention (formerly Neglect)**: Sufficient information to identify neglect may be obtained during the prior testing. If the patient has a severe visual loss preventing visual double simultaneous stimulation, and the cutaneous stimuli are normal, the score is normal. The standard diagram is shown to the patient and he is asked to describe it. The physician encourages the patient to look at the diagram carefully and identify the features on the left and right side of the diagram. If the patient cannot identify parts of the diagram on one side, it is designated as abnormal. Then, the doctor asks the patient to close his eyes and check bilateral skin sensation by measuring pinprick sensation in the upper or lower extremities, respectively. If the patient has neglected sensation on one side, it is abnormal.	0 = No abnormality.<br>1 = Visual, tactile, auditory, spatial, or personal inattention or extinction to bilateral simultaneous stimulation in one of the sensory modalities.<br>2 = Profound hemi-inattention or extinction to more than one modality; does not recognize own hand or orients to only one side of space.	____
**12**	**Additional non-NIHSS item Distal motor function**: The examiner holds the anterior portion of the patient's hand and instructs him/her to	0 = Normal (no flexion after 5 seconds).<br>1 = At least some stretch after 5 seconds, but not full extension;	Left: ____<br>Right: ____

	Instructions	Scale Definition	Score
	extend the fingers as far as possible. If the patient is unable or does not extend the fingers, the examiner fully extends the fingers and observes any flexion movements for 5 seconds. Only the first attempt is scored, and repeated instructions and trials are prohibited.	no score for any finger movement (no instruction given).<br>2 = No active stretching after 5 seconds; no score for finger movements at other times.	

Attachment

Figures for the 9th and 10th examinations

Testing Figure 1

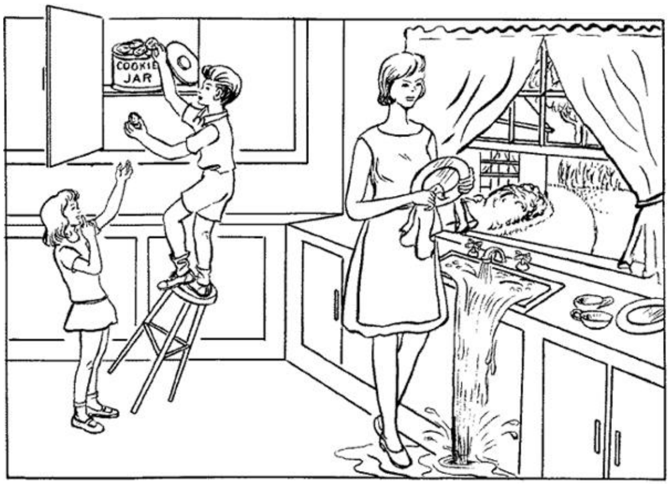

Testing Figure 2

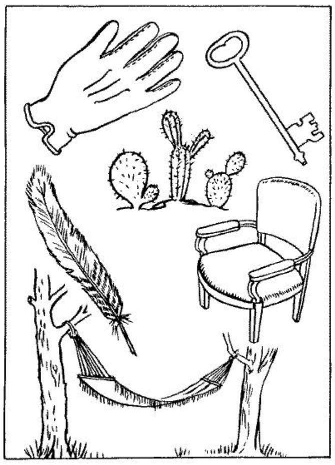

Testing Figure 3

Please read out the following sentences:

Know.

Go down the stairs.

Go home and cook.

Review at school.

Give a wonderful speech.

Testing Figure 4

Please read out the following words:

Mother

Earth

Plane

Silk

Start work on time.

Eat grapes without spitting grape skins.

How to evaluate the NIHSS score for coma patients

For patients with a score of less than 3 on 1a, each item should be rated individually. Item 1a is given a score of 3 only if the patient does not respond to any noxious stimulus (rubbing the sternum, pressing the orbit, etc.) and only has reflex activity. For patients scoring 3 on 1a, the remaining items should be scored as follows:

- ✧ Item 1b (LOC Questions): score 2
- ✧ Item 1c (LOC Commands): score 2
- ✧ Item 2 (Best Gaze): If it can be overcome by the head-eye reflex, score 1, if not, score 2.
- ✧ Item 3 (Visual): test using bilateral threat.

- Item 4 (Facial Palsy): score 3
- Item 5 and 6 (Motor Arm and Leg): score 4 for each limb.
- Item 7 (Limb Ataxia): scoring can be given only in the presence of ataxia. A score of 0 is given if the patient has decreased muscle strength and is unable to complete the finger-nose examination, heel-knee-shin examination, etc.
- Item 8 (Sensory): score 2
- Item 9 (Best Language): score 3
- Item 10 (Dysarthria): score 2
- Item 11 (Extinction and Inattention): score 2

How is the total NIHSS score calculated?

The following should not be counted into the total score:

- Items 5 and 6: "9 = Amputation or joint fusion" in limb movement.
- Item 7: The choice to determine the site of ataxia, i.e., "left upper limb 1 = Present in one limb. 2 = Present in two limbs. 9 = Amputation or joint fusion" (registration is not required).
- Additional Item 12 (registration is not required)

Annex 2: Modified Rankin Scale (mRS)

**Description**	**Rankin Grade**
No symptoms at all	0
No significant disability despite symptoms; able to carry out all usual duties and activities	1
Slight disability; unable to carry out all previous activities but able to look after own affairs without assistance	2
Moderate disability; requiring some help, but able to walk without assistance	3
Moderately severe disability; unable to walk without assistance, and unable to attend to own bodily needs without assistance	4
Severe disability: bedridden, incontinent, and requiring constant nursing care and attention	5

Evaluation of mRS

The modified Rankin Scale is used to measure the results of patients' functional recovery after stroke. **Bold** typeface shows the formal definition of each level. The ***italics*** give further guidance in order to reduce the possible errors between different observers, but there is no requirement for the structure of the interview. Please note that only symptoms that have occurred since the stroke are considered. If the patient can walk with the help of some assistive devices without outside help, it is considered to be able to walk independently.

If the two levels seem to be equally applicable to the patient, and further questions are unlikely to make an absolutely correct choice, the more severe level should be selected.

0- No symptoms at all

There may be mild symptoms. But no noticeable new functional limitations or new symptoms after stroke event.

1- No significant disability despite symptoms; able to carry out all usual duties and activities

The patient has some symptoms caused by the stroke, either physical or cognitive (e.g., affecting speech, reading, or writing; or body movement; or sensation; or vision; or swallowing; or emotion), but can continue to do all the work, social, and leisure activities that they did before the stroke. The key question used to distinguish levels 1 and 2 (see below) could be, "Are there things that you used to do regularly but could no longer do until after the stroke?". Activities that occur more frequently than once a month are considered "regular".

2- Slight disability; unable to carry out all previous activities but able to look after own affairs without assistance

Some activities that could be done before the stroke (such as driving, dancing, reading, or working) are no longer available to the person after the stroke, but the person is still able to care for themselves daily without assistance. Patients are able to dress, walk, eat, go to the bathroom, prepare simple foods, shop, and travel locally without assistance. The patient lives without supervision. It is envisioned that patients at this level can be home alone for a week or more without care.

3- Moderate disability; requiring some help, but able to walk without assistance

At this level, the patient is able to walk independently (with the aid of a walking aid) and can dress, go to the bathroom, eat, etc., but more complex tasks need to be completed with the assistance of others. For example, someone else is required to do the shopping, cooking, or cleaning, and to visit the patient more than once a week to ensure that the above activities are completed. Assistance is needed not only to take care of the body but also to give advice: for example, patients at this level will need supervision or encouragement to handle finances.

4- Moderately severe disability; unable to walk without assistance, and unable to attend to own bodily needs without assistance

Patients need other people to help with activities of daily living, whether it is

walking, dressing, going to the bathroom or eating. The patient requires care at least once a day, usually twice or more, or must live in close proximity to the caregiver. To distinguish levels 4 and 5 (see below), consider whether the patient is able to routinely live alone for an appropriate amount of time during the day.

5- Severe disability: bedridden, incontinent, and requiring constant nursing care and attention

Although trained nurses are not required, someone is needed to look after them several times throughout the day and night.

In this trial, death was attributed to a grade of 6.

Annex 3: Montreal Cognitive Assessment Scale (MoCA)

Name: ________ Gender: ________ Age: ________

Period of schooling (Year): ________ Date of assessment: ________________

**VISUOSPATIAL /EXECUTIVE** 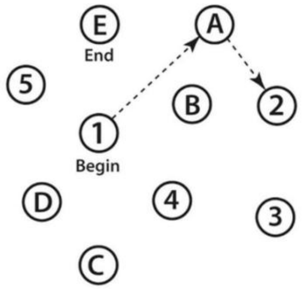	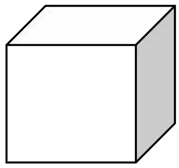	Draw CLOCK (Ten past eleven) (3 points)	**POINTS**
[ ]	[ ]	[ ] [ ] [ ] Contour Numbers Hands	__/5
**NAMING**			
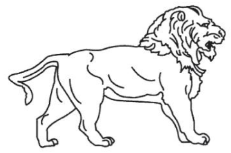	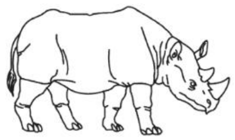	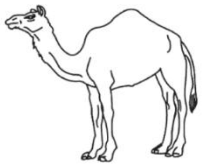	__/3
[ ]	[ ]	[ ]	

			FACE	VELVET	CHURCH	DAISY	RED	
**MEMORY**	Read list of words, subject must repeat them. Do 2 trials, even if 1st trial is successful. Do a recall after 5 minutes.	1ST TRIAL						NO POINTS
		2ND TRIAL						

**ATTENTION**	Read list of digits (1 digit/ sec.).	Subject has to repeat them in the forward order[ ]	**2 1 8 5 4**	__/2
		Subject has to repeat them in the backward order.[ ]	**7 4 2**	

Read list of numbers. The subject must tap with his hand at each number 1. No points if ≥ 2 errors.<br>[ ] 5 2 1 3 9 4 1 1 8 0 6 2 1 5 1 9 4 5 1 1 1 4 1 9 0 5 1 1 2	__/1

Serial 7 subtraction starting at 100.	[ ]93	[ ]86	[ ]79	[ ]72	[ ]65	__/3
4 or 5 correct subtractions: 3 pts, 2 or 3 correct: 2 pts, 1 correct: 1 pt, 0 correct: 0						

**LAN**	Repeat: I only know that Liang Zhang is the one to help today. [ ]	__/2

**GU AG E**	The cat always hid under the couch when dogs were in the room. [ ]							__/1
Fluency: Name as many animals as you can in one minute. [ ] ________(N≥11 words)								
**ABS TRA CTI ON**	Similarity between e.g. banana - orange = fruit [ ] train - bicycle [ ] watch - ruler							__/2
**DEL AYE D REC ALL**	Has to recall words WITH NO CUE	FACE [ ]	VELVE T [ ]	CHU RCH [ ]	DAIS Y [ ]	RED [ ]	Points for UNCUED recall only	__/5
Opti ons	Category cue							
	Multiple choice cue							
**ORI ENT ATI ON**	[ ] Date [ ] Month [ ] Year [ ] Day [ ] Place [ ] City							__/6
TOT AL	Add 1 point if ≤ 12 year education							__/30

Administration and Scoring Instructions

1.Alternating Trail Making

Administration: The examiner instructs the subject: "Please draw a line, going from a number to a letter in ascending order. Begin here [point to (1)] and draw a line from 1 then to A then to 2 and so on. End here [point to (E)]."

Scoring: Allocate one point if the subject successfully draws the following pattern: 1-A-2-B-3-C-4-D-5-E, without drawing any lines that cross. Any error that is not immediately self-corrected earns a score of 0.

2. Visual Constructional Skills (Cube)

Administration: The examiner gives the following instructions, pointing to the cube: "Copy this drawing as accurately as you can, in the space below."

Scoring: One point is allocated for a correctly executed drawing.

- ✧ Drawing must be three-dimensional
- ✧ All lines are drawn
- ✧ No line is added
- ✧ Lines are relatively parallel and their length is similar (rectangular prisms are accepted)

A point is not assigned if any of the above-criteria are not met.

3. Visual Constructional Skills (Clock)

Administration: Indicate the right third of the space and give the following instructions: "Draw a clock. Put in all the numbers and set the time to 10 after 11".

Scoring: One point is allocated for each of the following three criteria:

- ✧ Contour (1 pt.): the clock face must be a circle with only minor distortion acceptable (e.g., slight imperfection on closing the circle);
- ✧ Numbers (1 pt.): all clock numbers must be present with no additional numbers; numbers must be in the correct order and placed in the approximate quadrants on the clock face; Roman numerals are acceptable; numbers can be placed outside the circle contour;
- ✧ Hands (1 pt.): there must be two hands jointly indicating the correct time; the hour hand must be clearly shorter than the minute hand; hands must be centred within the clock face with their junction close to the clock centre.

A point is not assigned for a given element if any of the above-criteria are not met.

4. Naming

Administration: Beginning on the left, point to each figure and say: "Tell me the name of this animal."

Scoring: One point each is given for the following responses: (1) lion, (2) rhinoceros or rhino, (3) camel or dromedary.

5. Memory

Administration: The examiner reads a list of 5 words at a rate of one per second, giving the following instructions: "This is a memory test. I am going to read a list of words that you will have to remember now and later. Listen carefully. When I am through, tell me as many words as you can remember. It doesn't matter in what order you say them". Mark a check in the allocated space for each word the subject produces on this first trial. When the subject indicates that (s)he has finished (has recalled all words), or can recall no more words, read the list a second time with the following instructions: "I am going to read the same list for a second time. Try to remember and tell me as many words as you can, including words you said the first time." Put a check in the allocated space for each word the subject recalls after the second trial.

At the end of the second trial, inform the subject that (s)he will be asked to recall these words again by saying, "I will ask you to recall those words again at the end of the test."

Scoring: No points are given for Trials One and Two.

6. Attention

Forward Digit Span **Administration**: Give the following instruction: "I am going to say some numbers and when I am through, repeat them to me exactly as I said them". Read the five number sequence at a rate of one digit per second.

Backward Digit Span **Administration**: Give the following instruction: "Now I am going to say some more numbers, but when I am through you must repeat them to me in the backwards order." Read the five number sequence at a rate of one digit per second.

Scoring: Allocate one point for each sequence correctly repeated, (N.B.: the

correct response for the backwards trial is 2-4-7).

Vigilance **Administration**: The examiner reads the list of letters at a rate of one per second, after giving the following instruction: "I am going to read a sequence of letters. Every time I say the letter A, tap your hand once. If I say a different letter, do not tap your hand".

Scoring: Give one point if there is zero to one errors (an error is a tap on a wrong letter or a failure to tap on letter A).

Administration: The examiner gives the following instruction: "Now, I will ask you to count by subtracting seven from 100, and then, keep subtracting seven from your answer until I tell you to stop." Give this instruction twice if necessary.

Scoring: This item is scored out of 3 points. Give no (0) points for no correct subtractions, 1 point for one correction subtraction, 2 points for two-to-three correct subtractions, and 3 points if the participant successfully makes four or five correct subtractions. Count each correct subtraction of 7 beginning at 100. Each subtraction is evaluated independently; that is, if the participant responds with an incorrect number but continues to correctly subtract 7 from it, give a point for each correct subtraction. For example, a participant may respond "92 - 85 - 78 - 71 - 64" where the "92" is incorrect, but all subsequent numbers are subtracted correctly. This is one error and the item would be given a score of 3.

7. Sentence repetition

Administration: The examiner gives the following instructions: "I am going to read you a sentence. Repeat it after me, exactly as I say it [pause]: I only know that Liang Zhang is the one to help today." Following the response, say: "Now I am going to read you another sentence. Repeat it after me, exactly as I say it [pause]: The cat always hid under the couch when dogs were in the room."

Scoring: Allocate 1 point for each sentence correctly repeated. Repetition must be exact. Be alert for errors that are omissions (e.g., omitting "only", "always") and

substitutions/additions (e.g., "Liang Zhang is the one who helped today;" substituting "hides" for "hid", altering plurals, etc.).

8. Verbal fluency

Administration: The examiner gives the following instruction: "Tell me as many animals' names as you can think of. I will tell you to stop after one minute. Are you ready? [Pause] Now, tell me as many animals as you can think of. [time for 60 sec]. Stop."

Scoring: Allocate one point if the subject generates 11 animals or more in 60 sec. Record the subject's response in the bottom or side margins. Dragons, phoenixes, unicorns, and other deified animals are also considered correct.

9. Abstraction

Administration: The examiner asks the subject to explain what each pair of words has in common, starting with the example: "Tell me how an orange and a banana are alike". If the subject answers in a concrete manner (e.g., both have peels, or both can be eaten, etc.), then say only one additional time: "Tell me another way in which those items are alike". If the subject does not give the appropriate response (fruit), say, "Yes, and they are also both fruit." Do not give any additional instructions or clarification.

After the practice trial, say: "Now, tell me how a train and a bicycle are alike". Following the response, administer the second trial, saying: "Now tell me how a ruler and a watch are alike". Do not give any additional instructions or prompts.

Scoring: Only the last two item pairs are scored. Give 1 point to each item pair correctly answered.

The following responses are acceptable:

- ✧ Train-bicycle = means of transportation, means of travelling, you take trips in both.

✧ Ruler-watch = measuring instruments, used to measure.

The following responses are not acceptable:

✧ Train-bicycle = they have wheels.

✧ Ruler-watch = they have numbers.

10. Delayed recall

Administration: The examiner gives the following instruction: "I read some words to you earlier, which I asked you to remember. Tell me as many of those words as you can remember." Make a check mark (√) for each of the words correctly recalled spontaneously without any cues, in the allocated space.

Scoring: Allocate 1 point for each word recalled freely without any cues.

Optional:

Following the delayed free recall trial, prompt the subject with the semantic category cue provided below for any word not recalled. Make a check mark (√) in the allocated space if the subject remembered the word with the help of a category or multiple-choice cue. Prompt all non-recalled words in this manner. If the subject does not recall the word after the category cue, give him/her a multiple-choice trial, using the following example instruction, "Which of the following words do you think it was, NOSE, FACE, or HAND?"

Use the following category and/or multiple-choice cues for each word, when appropriate:

	Category cue	Multiple choice
FACE:	part of the body	nose, face, hand
VELVET:	type of fabric	denim, cotton, velvet
CHURCH:	type of building	church, school, hospital
DAISY:	type of flower	rose, daisy, tulip
RED:	a color	red, blue, green

Scoring: No points are allocated for words recalled with a cue. A cue is used

for clinical information purposes only and can give the test interpreter additional information about the type of memory disorder. For memory deficits due to retrieval failures, performance can be improved with a cue. For memory deficits due to encoding failures, performance does not improve with a cue.

11. Orientation

Administration: The examiner gives the following instructions: "Tell me the date today". If the subject does not give a complete answer, then prompt accordingly by saying: "Tell me the [year, month, exact date, and day of the week]." Then say: "Now, tell me the name of this place, and which city it is in."

Scoring: Give one point for each item correctly answered. The subject must tell the exact date and the exact place (name of hospital, clinic, office). No points are allocated if subject makes an error of one day for the day and date.

Total Score

Sum all sub-scores listed on the right-hand side. Add one point for an individual who has 12 years or fewer of formal education, for a possible maximum of 30 points. A final total score of 26 and above is considered normal.

Annex 4: EuroQol Five-Dimensions (EQ-5D) Health Scale

**Under each heading, please tick the ONE box that best describes your health TODAY.**	
**MOBILITY**	□1 I have no problems in walking about<br>□2 I have some problems in walking about<br>□3 I am confined to bed
**SELF-CARE**	□1 I have no problems with self-care<br>□2 I have some problems washing or dressing myself<br>□3 I am unable to wash or dress myself
**USUAL ACTIVITIES** (e.g., work, study, housework, family, or leisure activities)	□1 I have no problems with performing my usual activities<br>□2 I have some problems with performing my usual activities<br>□3 I am unable to perform my usual activities
**PAIN / DISCOMFORT**	□1 I have no pain or discomfort<br>□2 I have moderate pain or discomfort<br>□3 I have extreme pain or discomfort
**ANXIETY / DEPRESSION**	□1 I am not anxious or depressed<br>□2 I am moderately anxious or depressed<br>□3 I am extremely anxious or depressed
**We would like to know how good or bad your health is TODAY. This scale is numbered from 0 to 100. 100 means the best health you can imagine. 0 means the worst health you can imagine.**<br>**Please mark your health status today on the scale on the right. Please draw a line from the square below to the point on the scale that best represents your health today.**	

YOUR HEALTH TODAY = [ ]

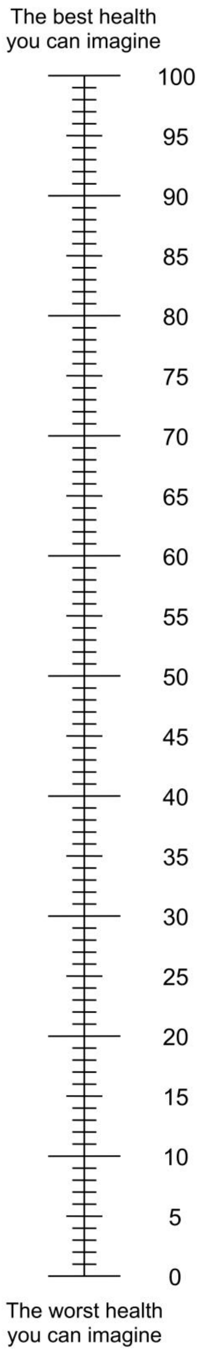

The Efficacy and Safety of Ginkgo Diterpene Lactone Meglumine in Acute Ischemic Stroke

Statistical Analysis Plan

Principal Investigator

Yongjun Wang, MD, PhD

Beijing Tiantan Hospital, Capital Medical University, Beijing, China

Prepared by

Anxin Wang, PhD

Xue Tian, PhD

Beijing Tiantan Hospital, Capital Medical University, Beijing, China

Version 1.0

Jan 1, 2016

Table of Contents

1. Introduction 91
2. Study Objectives 91
  2.1 Primary Objective 91
  2.2 Secondary Objectives 91
  2.3 Exploratory Objectives 92
3. Study Design 92
4. Study Measures 93
  4.1 Primary Efficacy Measure 93
  4.2 Secondary Efficacy Measure 93
  4.3 Exploratory Measure 94
  4.4 Safety Measure 94
5. Analysis Populations 95
  Full Analysis Set (FAS) 95
  Per Protocol Set (PPS) 95
  Safety Set (SS) 95
6. Sample Size Estimate 96
7. Statistical Analyses 96
  7.1 General Principles 96
    7.1.1 Test Level 96
    7.1.2 Hypothesis Test 96
    7.1.3 Missing Data 97
  7.2 Study Population 97
    7.2.1 Disposition of Patients 97
    7.2.2. Protocol Violations and Deviations 97
  7.3 Demographic and Baseline Characteristics 98
  7.4 Efficacy Analyses 98
    7.4.1 Primary Efficacy Analysis 98
    7.4.2 Secondary Efficacy Analyses 99
    7.4.3 Exploratory Efficacy Analyses 101

7.5 Safety Analysis 102
7.5.1 Extent of Exposure and Compliance 102
7.5.2 Adverse Events 102
7.5.3 Severe Adverse Events 103
7.5.4 Vital Signs and Laboratory Test Evaluation 103

1. Introduction

This statistical analysis plan (SAP) documents the planned statistical analyses for the trial and is based on the protocol, together with any subsequent amendments. This SAP is intended for the use of project team members and should be read in conjunction with the aforementioned protocol.

2. Study Objectives

2.1 Primary Objective

To evaluate the efficacy of ginkgo diterpenelactone meglumine injection in achieving a good functional outcome (mRS 0-1) on day 90 after randomization in patients with acute ischemic stroke.

2.2 Secondary Objectives

(1) To evaluate the difference in the proportion of mRS 0-2 on day 90 between the two groups;
(2) To evaluate the difference in the recovery of neurological deficits (decline in NIHSS score ≥4) on day 7 between the two groups;
(3) To evaluate the difference in the recovery of neurological deficits (decline in NIHSS score ≥4) on day 14 between the two groups;
(4) To evaluate the difference in deterioration of neurological deficits (increase in NIHSS score ≥4) on day 7 between the two groups;
(5) To evaluate the difference in deterioration of neurological deficits (increase in NIHSS score ≥3) on day 7 between the two groups;
(6) To evaluate the difference in deterioration of neurological deficits (increase in NIHSS score ≥2) on day 7 between the two groups;
(7) To evaluate the difference in deterioration of neurological deficits (increase in NIHSS score ≥1) on day 7 between the two groups;
(8) Subgroup analyses: the primary efficacy outcome will also be analyzed stratified by age (<65/≥65), sex (male/female), previous stroke (yes/no), hypertension (yes/no), diabetes mellitus (yes/no), time from onset to treatment (<24/≥24), and NIHSS score (≤7/>7);

(9) To compare the safety of two groups in term of:

- ✧ Difference in the adverse events;
- ✧ Difference in changes of vital signs from baseline to day 14;
- ✧ Difference in changes of laboratory variables from baseline to day 14, including blood routine (red blood cell, white blood cell count, hemoglobin, platelet count), urine routine (leucocyte, protein, glucose), liver function (alanine aminotransferase, aspartate transaminase, alkaline phosphatase, γ-glutamyl transpeptadase), and kidney function (urea nitrogen, creatinine).

2.3 Exploratory Objectives

(1) To evaluate the difference in the improvement of MoCA on day 14 between the two groups;

(1) To evaluate the difference in the improvement of MoCA on day 90 between the two groups;

(5) To evaluate the difference in the improvement of EQ-5D on day 14 between the two groups;

(5) To evaluate the difference in the improvement of EQ-5D on day 90 between the two groups;

(6) To evaluate the difference in the rate of all-cause mortality, recurrent ischemic stroke, and combined vascular event (including stroke, myocardial infarction, vascular death, and angina pectoris) within 90 days between the two groups;

3. Study Design

This study is a multicenter, double-blind, placebo-controlled, randomized clinical trial, which aims to evaluate the efficacy of ginkgo diterpenelactone meglumine injection in the treatment of acute ischemic stroke. Eligible criteria for the trial population include age between 18 and 80 years, within 48 hours of stroke onset of ischemic stroke, with mRS score ≤1 prior to onset, with NIHSS score between 4 and 24, have a total score of upper and lower limbs on motor deficits ≥2, and signinformed consent. Patients are not eligible if they have encephalitis, brain tumor, brain abscess and cause similar symptoms of disease, or confirm with hemorrhagic cerebral infarction, epidural hematoma, intracranial hematoma, intraventricular hemorrhage, subarachnoid hemorrhage, etc. confirmed by head imaging studies; have serious disturbance of consciousness (Ia NIHSS score 2 points or higher); have hemorrhagic disease or have a bleeding tendency, or have a lower limb

venous thrombosis; have serious abnormal liver and kidney function; have a history of mental illness or dementia; have severe organ or other systemic disease, accompanied by any organ or system of malignant tumor, or ongoing anti-tumor treatment, the estimated lifetime < 3 months; have significant drug or alcohol abuse; have allergic constitution, as well as to two or more drugs or food allergies, or have this medicine ingredients allergy or known; have pregnancy during the test preparation is pregnancy or lactation in women; have participated in other clinical trials during the past three months; unsuitable for this study in the opinion of the investigators. All eligible patients will be randomly assigned receive ginkgo diterpenelactone meglumine injection or placebo in a 1:1 ratio. Patients in the ginkgo diterpenelactone meglumine injection will receive ginkgo diterpenelactone meglumine injection 5ml once daily for 14 consecutive days. The placebo group received ginkgo diterpenelactone meglumine injection simulation 5ml once daily for 14 consecutive days. Both the ginkgo diterpenelactone meglumine injection and placebo will be diluted in 250 mL of sterile 0.9% sodium chloride injection. All the patients will be followed up to day 90 after the randomization. The trial was approved by the ethics committee at Beijing Tiantan Hospital and at each participating site. Written informed consent for participation in the trial was provided by the patients or their representatives. The study was registered at ClinicalTrials.gov (NCT02526225).

4. Study Measures

4.1 Primary Efficacy Measure

The proportion of mRS of 0-1 on day 90 after randomization.

- ✧ mRS scores range from 0 to 6, and score 0 indicates no symptoms, score 1 indicates no clinically significant disability, score 2 indicates slight disability, score 3 indicates moderate disability, score 4 indicates moderately severe disability, score 5 indicates severe disability, and score 6 indicates death.

4.2 Secondary Efficacy Measure

(1) The proportion of patients with mRS 0-2 on day 90;

(2) The proportion of patients with recovery of neurological deficits on day 7: decline in NIHSS score ≥4;

(3) The proportion of patients with recovery of neurological deficits on day 14: decline in NIHSS score ≥4;

(4) The proportion of patients with deterioration of neurological deficits on day 7: increase in NIHSS score increase in NIHSS score ≥4;

(5) The proportion of patients with deterioration of neurological deficits on day 7: increase in NIHSS score increase in NIHSS score ≥3;

(6) The proportion of patients with deterioration of neurological deficits on day 7: increase in NIHSS score increase in NIHSS score ≥2;

(7) The proportion of patients with deterioration of neurological deficits on day 7: increase in NIHSS score increase in NIHSS score ≥1;

4.3 Exploratory Measure

(1) Changes in MoCA from baseline to day 14;

(2) Changes in MoCA from baseline to day 90;

(3) Changes in Quality of Life (EuroQol EQ-5D scale) from baseline to day 14.

✧ The EQ-5D consists of five dimensions (mobility, self-care, usual activities, pain or discomfort, and anxiety or depression) and a subjectively rated visual analog scale. EQ-5D utility-scoring algorithms will be used from three conversion tables from the United Kingdom, the United States, and Japan, based on time trade-off models.

(4) Changes in Quality of Life (EuroQol EQ-5D scale) from baseline to day 90.

(5) The rate of all-cause mortality within 90 days;

(6) The rate of recurrent ischemic stroke within 90 days;

(7) The rate of combined vascular events within 90 days, including stroke, myocardial infarction, vascular death, and angina pectoris.

4.4 Safety Measure

(1) Adverse events within 90 days;

(2) Severe Adverse events within 90 days;

(2) Changes in vital signs and laboratory variables from baseline to day 14: blood pressure, heart rate, breathing, body temperature, blood routine (hemoglobin, platelet count, red blood cell, white blood cell count), urine routine (red blood cell count, white blood cell count, glucose, protein), and blood biochemistry (alkaline phosphatase, alanine aminotransferase, aspartate transaminase, γ-glutamyl transpeptidase, total bilirubin, urea nitrogen, creatinine).

5. Analysis Populations

Full Analysis Set (FAS)

According to the basic principle of modified Intention-to-teat (mITT) Analysis, all subjects who who had undergone randomization and had at least one assessment of efficacy after baseline. The last observation carry-forward (LOCF) estimation method can be used to estimate missing values. The mITT is the main validity evaluation population in this study.

Per Protocol Set (PPS)

Per Protocol Set (PPS) is a subset of FAS and includes all subjects who completed protocol-specified treatments or who did not have serious protocol violations. The exact definition of a serious violation of the protocol will be finalized at the time of data review, which may generally include (but is not limited to) the following situations: failure to meet the main inclusion criteria, the treatment that seriously interferes with efficacy evaluation after inclusion, poor compliance, and severe follow-up beyond the time window, etc. PPS is a secondary analysis population for validity, but if its results are inconsistent with the full analysis set, a detailed analysis of the inconsistent results is required. PPS includes subjects who completed study treatment without serious violation of protocol. The definition of serious violation against protocol will be confirmed at the data review.

Safety Set (SS)

All patients who received at least 1-time of study treatment according to the study protocol and safety assessment available will be included in the safety population. Throughout the safety results sections, erroneously treated patients (eg, those randomized to the experimental group but given

placebo) will be accounted for in the actual treatment group. This population will be used for safety analyses.

6. Sample Size Estimate

The study hypothesized that there is no difference in the rate of mRS of 0-1 on day 90.

According to the results of previous studies, it is assumed that the proportion of patients in the placebo group with mRS of 0-1 is 45%, the proportion of patients in the ginkgo diterpenelactone meglumine injection group with mRS of 0-1 is 50%. When the test level is 0.05, the power is 0.80, 1,565 patients are needed in each group. Considering an approximate 10% loss of follow-up, a total of 3,452 patients are needed in this study, with 1726 patients in each group. PASS11 (NCSS, LLC) will be used to calculate the sample size.

7. Statistical Analyses

7.1 General Principles

Biostatisticians and primary investigators formulate statistical analysis plans according to the research plan, and finalize the protocol before data locking. All programming will be performed using SAS Version 9.4.

For continuous variables, mean, median, standard deviation, maximum, minimum, $25^{th,}$ and 75th of the subjects that are not missing will be listed. For categorized or ordinal variables, frequency tables (frequencies and percentages) of the subjects that are not missing will be listed.

7.1.1 Test Level

All hypotheses will be tested by a two-sided type I error of 0.05. All statistics were two-sided with a P<0.05 considered significant. The reliability of all confidence intervals (CIs) will be 95%.

7.1.2 Hypothesis Test

The primary outcome of this study is the proportion of mRS 0-1 on day 90 after randomization. In this study, the null hypothesis of no difference in the rate of mRS 0-1 on day 90 between the two treatment groups will be tested using a two-sided test at the 5% level of significance.

H0: $\lambda_1=\lambda_2$

H1: $\lambda_1\neq\lambda_2$

Where λ_1 is the rate of mRS 0-1 on day 90 treated with ginkgo diterpenelactone meglumine injection and λ_2 is the same outcome in the placebo-controlled group.

7.1.3 Missing Data

Missing data on the primary efficacy outcome of mRS score will be filled in using LOCF. Missing data in the secondary efficacy outcomes will not estimate.

7.2 Study Population

7.2.1 Disposition of Patients

The number of subjects in each analysis population will be presented, subjects to be excluded from the Per Protocol population will be listed, and the total number of subjects attending each clinic visit will also be summarized. The number of subjects randomized, completed, and prematurely withdrawn from the study will be presented for each treatment group. The primary reasons for withdrawal both prior to and post randomization will also be presented. A data display listing and summary of deviations from the inclusion/exclusion criteria will be presented for all subjects who were either entered or randomized into the trial.

7.2.2. Protocol Violations and Deviations

Subject data will be examined for evidence of protocol violators to assess how well the protocol was followed. Inclusion and exclusion criteria are detailed in the study protocol.

Subjects who commit protocol violations will be included in the FAS Population butnot in the the Per Protocol Population. These protocol violations will be shown in a listing. Subjects can either be full or partial protocol violators. A full protocol violator is completely excluded from the Per Protocol Population. A partial protocol violator has only some data excluded. For subjects who violated the protocol during the treatment period due to unpermitted changes in the treatment or prohibited concurrent treatment, the analysis will only use data recorded prior to the violation. For

all violations which reference the treatment period, the treatment start date will be used as the reference date.

A listing of all possible protocol violators will be produced for clinical review. The final list of subjects who are protocol violators and are therefore excluded from the Per-Protocol population will be agreed by the study team.

7.3 Demographic and Baseline Characteristics

Demographic characteristics, including age, sex, ethnic, and body mass index, will be listed and summarized for subjects in each treatment group. This will also be done for medical history (including stroke, hypertension, and diabetes), and NIHSS score.

Vital signs, including systolic blood pressure, and diastolic blood pressure, will also be listed and summarized in each treatment group.

The continuous data followed by normal distribution will be presented as mean and standard deviation and the continuous data followed by skewness distribution will be presented as median and interquartile range; categorical data will be presented as n (%). T-test or Wilcoxon rank sum test will be used for comparison between two continuous data, and Chi-squared tests or Fisher exact test will be used for comparison between two categorical data.

7.4 Efficacy Analyses

7.4.1 Primary Efficacy Analysis

The primary outcome is the proportion of mRS of 0-1on day 90 after randomization. FAS will be the primary population for efficacy analyses. PPS will be used as the secondary population for the efficacy analyses. If the results in the PPS population are inconsistent with the FAS population, a detailed analysis of the inconsistent results is required.

The differences in the proportions of the primary outcome (mRS 0-1 on day 90) between treatment groups, and their corresponding 95% CIs, will be estimated based on Newcombe–Wilson. The primary outcome will be assessed with a logistic regression. The odds ratios (ORs) and 95% CIs will be reported.

Interactions with Subgroups

Summary tables will be produced for the predefined subgroups and interactions between treatment and these subgroups will be investigated, using a logistic regression model. A separate model will be used for each interaction to determine its significance. This will also be presented graphically on a forest plot.

The predefined subgroups include:

- Age (<65/≥65)
- Sex (male/female)
- Previous stroke (yes/no)
- Hypertension (yes/no)
- Diabetes mellitus (yes/no)
- Time from onset to treatment (<24/≥24)
- NIHSS score (≤7/>7)

7.4.2 Secondary Efficacy Analyses

(1) The proportion of patients with mRS 0-2 on day 90;

The differences in the proportions of patients with mRS 0-2 on day 90 between treatment groups, and their corresponding 95% CIs, will be estimated based on Newcombe–Wilson. The outcome will be assessed with a logistic regression. The odds ratios (ORs) and 95% confidence intervals (CIs) will be reported.

(2) The proportion of patients with recovery of neurological deficits on day 7: decline in NIHSS score ≥4;

The differences in the proportions of patients with recovery of neurological deficits between treatment groups, and their corresponding 95% CIs, will be estimated based on Newcombe–Wilson. The outcome will be assessed with a logistic regression. The ORs and 95% confidence CIs will be reported.

(3) The proportion of patients with recovery of neurological deficits on day 14: decline in NIHSS score ≥4;

The differences in the proportions of patients with recovery of neurological deficits between

treatment groups, and their corresponding 95% CIs, will be estimated based on Newcombe–Wilson. The outcome will be assessed with a logistic regression. The ORs and 95% confidence CIs will be reported.

(4) The proportion of patients with deterioration of neurological deficits on day 7: increase in NIHSS score ≥4;

The differences in the proportions of patients with deterioration of neurological deficits between treatment groups, and their corresponding 95% CIs, will be estimated based on Newcombe–Wilson. The outcome will be assessed with a logistic regression. The ORs and 95% confidence CIs will be reported.

(5) The proportion of patients with deterioration of neurological deficits on day 7: increase in NIHSS score ≥3;

The differences in the proportions of patients with deterioration of neurological deficits between treatment groups, and their corresponding 95% CIs, will be estimated based on Newcombe–Wilson. The outcome will be assessed with a logistic regression. The ORs and 95% confidence CIs will be reported.

(6) The proportion of patients with deterioration of neurological deficits on day 7: increase in NIHSS score ≥2;

The differences in the proportions of patients with deterioration of neurological deficits between treatment groups, and their corresponding 95% CIs, will be estimated based on Newcombe–Wilson. The outcome will be assessed with a logistic regression. The ORs and 95% confidence CIs will be reported.

(7) The proportion of patients with deterioration of neurological deficits on day 7: increase in NIHSS score ≥1;

The differences in the proportions of patients with deterioration of neurological deficits between treatment groups, and their corresponding 95% CIs, will be estimated based on Newcombe–Wilson. The outcome will be assessed with a logistic regression. The ORs and 95% confidence CIs will be reported.

7.4.3 Exploratory Efficacy Analyses

(1) Changes in MoCA from baseline to day 14;

Differences between treatment groups of changes in MoCA will be calculated as mean differences with 95% CIs by using a generalized linear model if the data are normal, and the t-test will be used for comparison between groups. If the data are skewed, the median differences and 95% CIs will be calculated by using a Hodges-Lehmann estimation, and a 2-sample Wilcoxon rank-sum test will be used for comparison between treatment groups.

(2) Changes in MoCA from baseline to day 90;

Differences between treatment groups of changes in MoCA will be calculated as mean differences with 95% CIs by using a generalized linear model if the data are normal, and the t-test will be used for comparison between groups. If the data are skewed, the median differences and 95% CIs will be calculated by using a Hodges-Lehmann estimation, and a 2-sample Wilcoxon rank-sum test will be used for comparison between treatment groups.

(3) Changes in Quality of Life (EuroQol EQ-5D scale) from baseline to day 14.

Differences between treatment groups of changes in the EQ-5D scale will be calculated as mean differences with 95% CIs by using a generalized linear model if the data are normal, and the t-test will be used for comparison between groups. If the data are skewed, the median differences and 95% CIs will be calculated by using a Hodges-Lehmann estimation, and a 2-sample Wilcoxon rank-sum test will be used for comparison between treatment groups.

(4) Changes in Quality of Life (EuroQol EQ-5D scale) from baseline to day 90.

Differences between treatment groups of changes in the EQ-5D scale will be calculated as mean differences with 95% CIs by using a generalized linear model if the data are normal, and the t-test will be used for comparison between groups. If the data are skewed, the median differences and 95% CIs will be calculated by using a Hodges-Lehmann estimation, and a 2-sample Wilcoxon rank-sum test will be used for comparison between treatment groups.

(5) The rate of all-cause mortality within 90 days;

The rate of all-cause mortality within 90 days will be analyzed using a Cox proportional hazard model. The hazard ratio with 95% CI will be reported.

(6) The rate of recurrent ischemic stroke within 90 days;

The rate of recurrent ischemic stroke within 90 days will be analyzed using a Cox proportional hazard model. The hazard ratio with 95% CI will be reported.

(7) The rate of combined vascular events within 90 days, including stroke, myocardial infarction, vascular death, and angina pectoris.

The rate of combined vascular events within 90 days will be analyzed using a Cox proportional hazard model. The hazard ratio with 95% CI will be reported.

7.5 Safety Analysis

All analyses of safety data will be carried out using the safety set (SS) population.

7.5.1 Extent of Exposure and Compliance

Study duration (days) = (end date of study - start date of study) + 1

Duration of treatment (days) = (end date of study treatment - start date of study treatment) + 1

Compliance = actual dose / planned dosage × 100%. If the compliance is between 70% and 120%, the compliance is good.

7.5.2 Adverse Events

Adverse event data will be coded according to the current version of MedDRA. Treatment Emergent Adverse Event (TEAE) will be used in the analysis, while adverse events that start prior to the first dose of study medication (pre-treatment) will not be involved into the safety analysis. Adverse events on study medication (during treatment) and after the last dose of study medication (post-treatment) will be analyzed.

The differences in the proportions of patients with AE between treatment groups and their corresponding 95% CIs will be estimated based on Newcombe–Wilson. The ORs and 95% CIs will be estimated with the logistic regression. Chi-squared test or Fisher's Exact test will be used to compare the number of each AE combination according to system organ class and preferred term between treatment groups.

7.5.3 Severe Adverse Events

The differences in the proportions of patients with SAE between treatment groups and their corresponding 95% CIs will be estimated based on Newcombe–Wilson. The ORs and 95% CIs will be estimated with the logistic regression. Chi-squared test or Fisher's Exact test will be used to compare the number of each SAE combination according to system organ class and preferred term between treatment groups.

7.5.4 Vital Signs and Laboratory Test Evaluation

Within each treatment group, the number and percentage of subjects with elevated in vital signs from baseline to day 14 after randomization will be described and Fisher's Exact test will be used to compare the difference between treatment groups. The baseline measurements and changes after treatment of blood routine and bloodbiochemistry will be described as "abnormal and clinically significant" will be determined by the researchers. Within each treatment group, the number and percentage of subjects with an "abnormal and clinically significant" value will be summarized and Fisher's Exact test will be used to compare the difference between treatment groups.
