# Supplementary material for: Efficacy and Safety of Ginkgo Diterpene Lactone Meglumine in Acute Ischemic Stroke: A Randomized Clinical Trial
Source: JAMA Netw Open. 2023 Aug 14;6(8):e2328828. doi: 10.1001/jamanetworkopen.2023.28828 (PMC10425831; doi:10.1001/jamanetworkopen.2023.28828)
Supplement: Supplement 2. — eTable 1. Inclusion and Exclusion Criteria eTable 2. Concomitant Treatment Within 90 Days eTable 3. Concomitant Prohibited Medication Used Within 90 Days eTable 4. Sensitivity Analyses eTable 5. Primary and Secondary Outcomes in Per-protocol Population eTable 6. Adverse Events Recorded During 90 Days of Follow-up eTable 7. Severe Adverse Events (by System Organ Class) During 90 Days of Follow-up eTable 8. Number of Patients with Clinically Significant Changes in Vital Signs and Laboratory Data on Day 14 of Follow-up [file jamanetwopen-e2328828-s002.pdf]

Supplemental Online Content

Zhang Q, Wang A, Xu Q, et al; GDLM group. Efficacy and safety of ginkgo diterpene lactone meglumine in acute ischemic stroke: a randomized clinical trial. *JAMA Netw Open.* 2023;6(8):e2328828. doi:10.1001/jamanetworkopen.2023.28828

eTable 1. Inclusion and Exclusion Criteria

eTable 2. Concomitant Treatment Within 90 Days

eTable 3. Concomitant Prohibited Medication Used Within 90 Days

eTable 4. Sensitivity Analyses

eTable 5. Primary and Secondary Outcomes in Per-protocol Population

eTable 6. Adverse Events Recorded During 90 Days of Follow-up

eTable 7. Severe Adverse Events (by System Organ Class) During 90 Days of Follow-up

eTable 8. Number of Patients with Clinically Significant Changes in Vital Signs and Laboratory Data on Day 14 of Follow-up

This supplemental material has been provided by the authors to give readers additional information about their work.

© 2023 Zhang Q et al. *JAMA Network Open.*

eTable 1. Inclusion and Exclusion Criteria.

Inclusion Criteria

- Aged 18 to 80 years of age;
- Within 48 hours of stroke onset of ischemic stroke (diagnosis standard by the Chinese medical association of the fourth national conference on cerebrovascular disease);
- The first onset, or always not obvious legacy of stroke sequela (mRS acuities were before the onset of 1);
- The degree of nerve function defect score (NIHSS) score 4 to 24 points, body movement component (NIHSS score paragraphs 5 and 6) total score 2 points or higher;
- Understand and voluntarily signed informed consent.

Exclusion Criteria

- Head imaging studies have confirmed that, encephalitis, brain tumor, brain abscess and cause similar symptoms of disease, or confirm with hemorrhagic cerebral infarction, epidural hematoma, intracranial hematoma, intraventricular hemorrhage, subarachnoid hemorrhage, etc.
- The serious disturbance of consciousness (Ia NIHSS score 2 points or higher);
- With hemorrhagic disease or have a bleeding tendency, or have a lower limb venous thrombosis;
- Serious abnormal liver and kidney function, liver function laboratory indexes of ALT > 3 ULN, renal laboratory ULN Cr > 1.5);
- A history of mental illness or dementia patients;
- Severe organ or other systemic disease, accompanied by any organ or system of malignant

© 2023 Zhang Q et al. *JAMA Network Open.*

tumor, or ongoing anti-tumor treatment, the estimated lifetime < 3 months;

- Significant drug or alcohol abuse;
- Allergic constitution, as well as to two or more drugs or food allergies; This medicine ingredients allergy or known;
- Have pregnancy (check blood HCG positive screening tests, namely HCG > 5 miu/mL), during the test preparation is pregnancy or lactation in women;
- In the past three months in other clinical trials;
- Researchers do not determine poor adherence, or any other suitable for patients to participate in this study.

Abbreviations: ALT, alanine transaminase; Cr, creatinine; HCG, human chorionic gonadatropin; mRS, modified Rankin Scale; NIHSS, National Institutes of Health Stroke Scale.

© 2023 Zhang Q et al. *JAMA Network Open.*

eTable 2. Concomitant Treatment within 90 Days.

Concomitant medication, n (%)	GDLM group (N=1725)	Placebo group (N=1723)
Antihypertensive	736 (42.7)	714 (41.4)
Antidiabetic	367 (21.3)	344 (20.0)
Lipid-lowering	1017 (59.0)	1029 (59.7)
Antiplatelet	1560 (90.4)	1534 (89.0)

Abbreviation: GDLM, Ginkgo diterpene lactone meglumine

© 2023 Zhang Q et al. *JAMA Network Open.*

eTable 3. Concomitant Prohibited Medication Used within 90 Days

Concomitant prohibited medication, n (%)	GDLM group (N=1725)	Placebo group (N=1723)
Neuroprotective agents and thrombolytic drugs	44 (2.6)	55 (3.2)
Edaravone	19 (1.1)	27 (1.6)
Nimodipine	17 (1.0)	17 (1.0)
Gangliosides	3 (0.2)	2 (0.1)
Cytidylcholine	0 (0.0)	1 (0.1)
Cerebroflucan or piracetam	8 (0.5)	7 (0.4)
Eurefrin or human urokinase	0 (0.0)	4 (0.2)
Brain activator or brain protein hydrolysate	0 (0.0)	2 (0.1)
Calf serum deprotein	0 (0.0)	1 (0.1)
Calf blood deprotein	1 (0.1)	1 (0.1)
Preparations of traditional Chinese medicine ingredients for the treatment of cerebral infarction	33 (1.9)	39 (2.3)
Chuanxiongzine	3 (0.2)	7 (0.4)
Salvia miltiorrhiza	4 (0.2)	8 (0.5)
Compound Salvia	2 (0.1)	1 (0.1)
Escin Sodium	1 (0.1)	3 (0.2)
Puerarin	2 (0.1)	1 (0.1)
Angelica	1 (0.1)	3 (0.2)
Safflower	2 (0.1)	0 (0.0)
Ginkgo Damo	0 (0.0)	1 (0.1)
Kudiezi	2 (0.1)	0 (0.0)
Xingnaojing	6 (0.3)	4 (0.2)

© 2023 Zhang Q et al. *JAMA Network Open.*

Xuesaitong	4 (0.2)	1 (0.1)
Shuxuetong	6 (0.3)	9 (0.5)
Naoxuekang	1 (0.1)	2 (0.1)
Naoxintong	1 (0.1)	1 (0.1)
Breviscapine	0 (0.0)	1 (0.1)
Xueshuantong	3 (0.2)	4 (0.2)

Abbreviation: GDLM, Ginkgo diterpene lactone meglumine

© 2023 Zhang Q et al. *JAMA Network Open.*

eTable 4. Sensitivity analyses

Primary outcome	GDLM group (N=1725)	Placebo group (N=1723)	Risk difference (95% CI), %	Odds ratio (95% CI)	Relative risk (95% CI)	P value
Multiple imputation*	910 (52.8)	783 (45.4)	7.31 (3.97 to 10.62)	1.34 (1.17 to 1.53)	1.16 (1.08 to 1.24)	<.001

Abbreviations: CI, confidence interval; NIHSS, National Institute of Health stroke scale.

*Imputed with multiple imputation under the assumption of missing at random. Variables in the model included all baseline characteristics

© 2023 Zhang Q et al. *JAMA Network Open.*

eTable 5. Primary and Secondary Outcomes in Per-protocol Population.

Outcome	GDLM group (N=1547)	Placebo group (N=1528)	Risk difference (95% CI), %	Odds ratio (95% CI)	Relative risk (95% CI)	*P* value
**Primary outcome**						
mRS score at day 90 ≤ 1, n (%)	816 (52.7)	688 (45.0)	7.72 (4.19 to 11.23)	1.36 (1.18 to 1.57)	1.17 (1.09 to 1.26)	<.001
**Secondary outcomes**					1.21 (1.16 to 1.26)	
mRS score at day 90 ≤ 2, n (%)	1334 (86.2)	1090 (71.3)	14.90 (12.04 to 17.73)	2.52 (2.10 to 3.02)	1.08 (0.96 to 1.22)	<.001
Changes of NIHSS score from baseline to day 7 ≤ -4, n (%)	421 (27.2)	385 (25.2)	2.02 (-1.09 to 5.12)	1.11 (0.95 to 1.30)	1.22 (1.15 to 1.31)	.20
Changes of NIHSS score from baseline to day 14 ≤ -4, n (%) [*]	949 (61.3)	765 (50.1)	11.25 (7.74 to 14.71)	1.58 (1.37 to 1.82)	1.21 (0.50 to 2.90)	<.001
Changes of NIHSS score from baseline to day 7 ≥ 4, n (%)	11 (0.7)	9 (0.6)	0.12 (-0.49 to 0.75)	1.21 (0.50 to 2.93)	1.06 (0.51 to 2.18)	.67
Changes of NIHSS score from baseline to day 7 ≥ 3, n (%)	15 (1.0)	14 (0.9)	0.05 (-0.67 to 0.78)	1.06 (0.51 to 2.20)	1.02 (0.61 to 1.73)	.88
Changes of NIHSS score from baseline to day 7 ≥ 2, n (%)	28 (1.8)	27 (1.8)	0.04 (-0.92 to 1.01)	1.02 (0.60 to 1.75)	0.63 (0.43 to 0.91)	.93
Changes of NIHSS score from baseline to day 7 ≥ 1, n (%)	44 (2.8)	69 (4.5)	-1.67 (-3.04 to -0.34)	0.62 (0.42 to 0.91)	1.01 (0.86 to 1.18)	.01

© 2023 Zhang Q et al. *JAMA Network Open.*

**Safety outcomes**					1.54 (0.67 to 3.54)	
Adverse events, n (%)	247 (16.2)	252 (16.3)	0.12 (-2.49 to 2.73)	1.01 (0.83 to 1.22)	1.17(1.09 to 1.26)	.93
Severe adverse events, n (%)	9 (0.6)	14 (0.9)	0.32 (-0.32 to 0.99)	1.54 (0.67 to 3.57)	1.21 (1.16 to 1.26)	.31

Abbreviations: CI, confidence interval; GDLM, Ginkgo diterpene lactone meglumine; mRS, modified Rankin Scale; NIHSS, National Institute of Health stroke scale.

* The number of patients with missing data was similar in the two treatment groups, missing data on NIHSS score on day 14 occurred in 0 patients in the GDLMgroup and 1 patient in the placebo group.

© 2023 Zhang Q et al. *JAMA Network Open.*

eTable 6. Adverse Events Recorded During 90 Days of Follow-up.

Variables	GDLM group (N=1725)	Placebo group (N=1723)	*P* value
Blood and lymphatic system disorders	23 (1.3)	19 (1.1)	.54
Cardiac disorders	5 (0.3)	8 (0.5)	.40
Ear and labyrinth disorders	2 (0.1)	0 (0.0)	.16
Endocrine disorders	20 (1.2)	15 (0.9)	.40
Eye disorders	4 (0.2)	2 (0.1)	.41
Gastrointestinal disorders	46 (2.7)	42 (2.4)	.67
General disorders and administration site conditions	6 (0.3)	9 (0.5)	.44
Hepatobiliary disorders	81 (4.7)	93 (5.4)	.35
Immune system disorders	0 (0.0)	4 (0.2)	.05
Infections and infestations	18 (1.0)	24 (1.4)	.35
Injury, poisoning and procedural complications	1 (0.1)	0 (0.0)	.32
Musculoskeletal and connective tissue disorders	9 (0.5)	6 (0.3)	.44
Nervous system disorders	44 (2.6)	42 (2.4)	.83
Renal and urinary disorders	49 (2.8)	53 (3.1)	.68
Reproductive system and breast disorders	3 (0.2)	2 (0.1)	.66
Respiratory, thoracic and mediastinal disorders	31 (1.8)	34 (2.0)	.70
Skin and subcutaneous tissue disorders	26 (1.5)	13 (0.8)	.04
Vascular disorders	6 (0.3)	6 (0.3)	1.00

© 2023 Zhang Q et al. *JAMA Network Open.*

Abbreviation: GDLM, Ginkgo diterpene lactone meglumine

* Fisher exact test.

© 2023 Zhang Q et al. *JAMA Network Open.*

eTable 7. Severe Adverse Events (by System Organ Class) During 90 Days of Follow-up.

Variables	GDLM group (N=1725)	Placebo group (N=1723)	*P* value
Infections and infestations	1 (0.1)	1 (0.1)	1.00*
Nervous system disorders	15 (0.9)	14 (0.8)	.85
Injury, poisoning and procedural complications	2 (0.1)	1 (0.1)	1.00*
Musculoskeletal and connective tissue disorders	0 (0.0)	1 (0.1)	.50*
Surgical and medical operations	2 (0.1)	1 (0.1)	1.00*
Respiratory, thoracic, and mediastinal disorders	3 (0.2)	1 (0.1)	.62*
Psychiatric disorders	1 (0.1)	0 (0.0)	1.00*
Benign, malignant, and unknown neoplasms	2 (0.1)	0 (0.0)	.50*
Immune system diseases	0 (0.0)	1 (0.1)	.50*
Gastrointestinal disorders	0 (0.0)	1 (0.1)	.50*
Blood and lymphatic system disorders	1 (0.1)	2 (0.1)	.62*
Disordersardiac disorders	1 (0.1)	0 (0.0)	1.00*

Abbreviation: GDLM, Ginkgo diterpene lactone meglumine

* Fisher exact test.

© 2023 Zhang Q et al. *JAMA Network Open.*

eTable 8. Number of Patients with Clinically Significant Changes in Vital Signs and Laboratory Data on Day 14 of Follow-up

Variables	GDLM group (N=1547)	Placebo group (N=1528)	*P* value
**Vital Signs**			
Systolic blood pressure increased	381 (23.7)	361 (22.5)	.39
Diastolic blood pressure increased	420 (26.2)	419 (26.1)	.95
Heart rate increased	684 (42.6)	700 (43.5)	.61
Respiratory rate increased	379 (23.6)	364 (22.6)	.50
Temperature increased	669 (41.7)	647 (40.2)	.39
**Laboratory Data**			
Erythrocyte	121 (8.2)	120 (8.1)	.92
White blood cell	78 (5.3)	91 (6.1)	.31
Hemoglobin	123 (8.3)	116 (7.8)	.62
Platelets	62 (4.2)	82 (5.5)	.09
Urine erythrocyte	66 (5.5)	50 (4.1)	.10
Urine leukocyte	70 (5.9)	72 (5.9)	1.00
Urine glucose	35 (2.9)	40 (3.3)	.64
Urine protein	39 (3.3)	44 (3.6)	.66
Alanine transaminase	183 (12.5)	173 (11.7)	.52
Aspartate aminotransferase	123 (8.5)	122 (8.4)	.91
Total bilirubin	47 (3.3)	42 (2.9)	.53
Alkaline phosphatase	66 (4.9)	65 (4.7)	.80

© 2023 Zhang Q et al. *JAMA Network Open.*

Glutamyl transaminase	66 (4.7)	79 (5.6)	.32
Urea nitrogen	75 (5.2)	82 (5.6)	.61
Creatinine	75 (5.2)	85 (5.8)	.45

Abbreviations: CI, confidence interval; GDLM, Ginkgo diterpene lactone meglumine.

© 2023 Zhang Q et al. *JAMA Network Open.*
