# Supplementary material for: Efficacy and Safety of Ginkgo Diterpene Lactone Meglumine in Acute Ischemic Stroke: A Randomized Clinical Trial
Source: JAMA Netw Open. 2023 Aug 14;6(8):e2328828. doi: 10.1001/jamanetworkopen.2023.28828 (PMC10425831; doi:10.1001/jamanetworkopen.2023.28828)
Supplement: Supplement 3. — Nonauthor Collaborators [file jamanetwopen-e2328828-s003.pdf]

Supplemental Online Content: Nonauthor Collaborators
*First name, last name, and suffix (if applicable) are required and will appear in PubMed.

***Group Name(s): GDLM group**

*First Name and Middle Initial(s)	*Last Name	*Suffix (eg, Jr, III)	Academic Degrees	Institution	Location (city, state/province, country)	Role or Contribution, eg, chair, principal investigator	Group (if more than 1 Group listed in the byline) and/or Subgroup (eg, Steering Committee)
Yongjun	Wang			Beijing Tiantan Hospital		Steering Committee, Executive Committee	
Maolin	He					Steering Committee	
Qiang	Dong					Steering Committee	
Gang	Zhao					Steering Committee	
Kangning	Chen					Steering Committee	
Peng	Xie					Steering Committee	
Yun	Xu					Steering Committee	
Yi	Yang					Steering Committee	
Li	Guo					Steering Committee	
Yansheng	Li					Steering Committee	
Yilong	Wang					Executive Committee	
Xiaoli	Zhang					Statistical and Data Management Center	
Anding	Xu					Clinical Event Adjudication Committee	
Yansheng	Li					Clinical Event Adjudication Committee	
Xudong	Pan					Clinical Event Adjudication Committee	
Cuilan	Xiao					Independent Medical Monitor	
David	Wang					Data and Safety Monitoring Board	
Hao	Li					Data and Safety Monitoring Board	
Ying	Gao					Data and Safety Monitoring Board	
Jielai	Xia					Independent Statistician	
Hongjian	Zhao			Affiliated Hospital of Chengdu University		Data collection	
Huijuan	Wang			Affiliated Hospital of Chengdu University		Data collection	
Yue	He			Affiliated Hospital of Jiangsu University		Data collection	
Weili	Li			Affiliated Hospital of Weifang Medical College		Data collection	
Xiangyang	Wei			Affiliated Hospital of Logistics University of Chinese People's Armed Police Forces		Data collection	
Junqing	Bai			Anshan Cancer Hospital		Data collection	
Shiwei	Cao			Anshan Central Hospital		Data collection	
Ying	Song			Baoding First Hospital		Data collection	
Zhanhu	Ma			Baoding First Hospital		Data collection	
Na	Zhang			Central Hospital Affiliated to Shenyang Medical College		Data collection	
Nianzhen	Yin			Chenzhou First People's Hospital		Data collection	
Long	Zeng			Chenzhou First People's Hospital		Data collection	
Likun	Zhang			Daqing People's Hospital		Data collection	
Fang	Yang			Emergency General Hospital		Data collection	

Supplemental Online Content: Nonauthor Collaborators
*First name, last name, and suffix (if applicable) are required and will appear in PubMed.

*First Name and Middle Initial(s)	*Last Name	*Suffix (eg, Jr, III)	Academic Degrees	Institution	Location (city, state/province, country)	Role or Contribution, eg, chair, principal investigator	Group (if more than 1 Group listed in the byline) and/or Subgroup (eg, Steering Committee)
Jing	Zhang			General Hospital of Hegang Mining Group		Data collection	
Jian	Wang			Guangdong Provincial People's Hospital Ganzhou Hospital (Ganzhou Municipal Hospital)		Data collection	
Huili	Tang			Guihang Guiyang Hospital		Data collection	
Yinghua	Li			Guizhou Aerospace Hospital		Data collection	
Hourong	Zhou			Guizhou Provincial People's Hospital		Data collection	
Haisong	Feng			Hanyang Hospital Affiliated to Wuhan University of Science and Technology		Data collection	
Dongjing	Song			Harbin Second Hospital		Data collection	
Jianyu	Wang			Harrison International Peace Hospital		Data collection	
Yongcui	Ma			Hegang People's Hospital		Data collection	
Lihui	Chen			Heilongjiang Nongken General Hospital		Data collection	
Kaiwen	Xiao			Hengyang First People's Hospital		Data collection	
Qijin	Zhai			Huaian First People's Hospital		Data collection	
Jing	Li			Huainan First People's Hospital		Data collection	
Xiaojie	Li			Inner Mongolia Baogang Hospital		Data collection	
Yinqiao	Yang			Jingmen No. 2 People’s Hospital		Data collection	
Xue	Mei			Jining First People's Hospital		Data collection	
Ziyun	Zhu			Jiujiang People's Hospital		Data collection	
Yan	Zhang			Kaifeng Central Hospital		Data collection	
Yuhu	Liu			Lanzhou University Second Hospital		Data collection	
Hongxia	Nie			Lianyungang Second People's Hospital		Data collection	
Bing	Fu			Lianyungang Second People's Hospital		Data collection	
Gang	Huang			Luohe Central Hospital		Data collection	
Guoli	Zeng			Luzhou People's Hospital		Data collection	
Bingrong	Liu			Maanshan Central Hospital		Data collection	
Wang	Xiaomin			Nanshi Hospital of Nanyang		Data collection	
Lianying	Xu			Nanyang Second People's Hospital		Data collection	
Fan	Zhang			Nuclear Industry 215 Hospital of Shaanxi Province		Data collection	
Lan	Wang			Qiqihar First Hospital		Data collection	
Wei	Han			Red Cross Hospital of Heilongnjiang Province		Data collection	
Shanshan	Meng			Shangqiu First People's Hospital		Data collection	
Chang'e	Xiao			Shaoyang First People's Hospital		Data collection	
Zhijie	Yang			Shengli Oilfield Central Hospital		Data collection	
Yanqing	Deng			Shiyan Renmin Hospital		Data collection	
Hu	Ying			Peking Universtiy Shougang Hospital		Data collection	

Supplemental Online Content: Nonauthor Collaborators
*First name, last name, and suffix (if applicable) are required and will appear in PubMed.

*First Name and Middle Initial(s)	*Last Name	*Suffix (eg, Jr, III)	Academic Degrees	Institution	Location (city, state/province, country)	Role or Contribution, eg, chair, principal investigator	Group (if more than 1 Group listed in the byline) and/or Subgroup (eg, Steering Committee)
Li	Tang			Sichuan University West China Hospital		Data collection	
Chunli	Jia			Suihua First People's Hospital		Data collection	
Chunpeng	Li			Suzhou Jiulong Hospital Affiliated to Shanghai Jiaotong University		Data collection	
Shihua	Liu			Suzhou Municipal Hospital		Data collection	
Min	Han			Taihe County People's Hospital		Data collection	
Yanhua	Zhang			Taiyuan Central Hospital		Data collection	
Feifei	Peng			Taizhou First People's Hospital		Data collection	
Jing	Zhao			The 169th Hospital of the Chinese People's Liberation Army		Data collection	
Donghai	Qiao			The 211th Hospital of the Chinese People's Liberation Army		Data collection	
Wenjing	Li			The 401th Hospital of the Chinese People's Liberation Army		Data collection	
Shengnan	Cai			The 463th Hospital of the Chinese People's Liberation Army		Data collection	
Qiuying	Song			The Affiliated Hospital of Hangzhou Normal University		Data collection	
Chenghua	Xiao			The Affiliated Hospital of Xuzhou Medical University		Data collection	
Ming	Chen			The Fifth Affiliated Hospital of Xinjiang Medical University		Data collection	
Junjie	Lei			The Fifth Afiliated Hospital,Sun Yat-sen University		Data collection	
Aimin	Cai			The First Affifiliated Hospital of Jinzhou Medical University		Data collection	
Wenfang	Xu			The First Affiliated Hospital of Bengbu Medical College		Data collection	
Jie	Cao			The First Affiliated Hospital of Gannan Medical College		Data collection	
Xinxin	Liu			The First Affiliated Hospital of Henan University		Data collection	
Dong	Tan			The First Affiliated Hospital of Hunan Medical College		Data collection	
Tao	Feng			The First Affiliated Hospital of Nanyang Medical College		Data collection	
Feng	Lina			The First Affiliated Hospital, School of Medicine, Shihezi University		Data collection	

Supplemental Online Content: Nonauthor Collaborators
*First name, last name, and suffix (if applicable) are required and will appear in PubMed.

***First Name and Middle Initial(s)**	***Last Name**	***Suffix (eg, Jr, III)**	Academic Degrees	Institution	Location (city, state/province, country)	Role or Contribution, eg, chair, principal investigator	Group (if more than 1 Group listed in the byline) and/or Subgroup (eg, Steering Committee)
Hongjing	Yan			The First Hospital of Handan City		Data collection	
Gulinazi				The First Hospital of Xinjiang Medical University		Data collection	
Changxia	Liu			The First People's Hospital of Yancheng City		Data collection	
Yao	Wang			The Fourth Hospital of Harbin Medical University		Data collection	
Qinghua	Yang			The Second Affiliated Hospital of Guizhou Medical University		Data collection	
Lin	Ji			The Second Affiliated Hospital of Shandong University of Chinese Medicine		Data collection	
Jing	Xu			The Second Affiliated Hospital of Xuzhou Medical College		Data collection	
Liping	Chen			The Second Hospital of Hebei Medical University		Data collection	
Shuai	Chen			The Second Hospital of Zhengzhou University		Data collection	
Yanfeng	Song			The Second People’s Hospital of LiaoCheng		Data collection	
Li	Mei			The Second People's Hospital of Guiyang (Jinyang Hospital)		Data collection	
Sheng	Ouyang			The Third Affiliated Hospital of Gansu University of Traditional Chinese Medicine, Gansu Province		Data collection	
Jie	Li			The Third Affiliated Hospital of Southern Medical University		Data collection	
Defu	Zhao			The Third Hospital of Liaoning Medical College		Data collection	
Zhaozuo	Dong			Tianjin People's Hospital		Data collection	
Wei	Jin			Tongling City People's Hospital		Data collection	
Guoxiang	Tang			Tongling Municipal Hospital		Data collection	
Na	Geng			Weihai Municipal Hospital		Data collection	
Hongxing	Cai			Wuhu First People's Hospital		Data collection	
Zaewang	Li			Wuxi People's Hospital		Data collection	
Yu	Chen			Xiangyang Central Hospital		Data collection	
Li	Huang			Xiangyang First People's Hospital Affiliated to		Data collection	
Yuzhang	Zhang			Yan'an University Xianyang Hospital		Data collection	
Peng	Lei			Yichang Central People's Hospital		Data collection	
Mingmin	Yan			Yichang First People's Hospital		Data collection	
Xiaojing	Zhang			Yuanping First People's Hospital		Data collection	
Yiqi	Wang			Zhejiang Provincial People's Hospital		Data collection	

Supplemental Online Content: Nonauthor Collaborators
*First name, last name, and suffix (if applicable) are required and will appear in PubMed.

***First Name and Middle Initial(s)**	***Last Name**	***Suffix (eg, Jr, III)**	Academic Degrees	Institution	Location (city, state/province, country)	Role or Contribution, eg, chair, principal investigator	Group (if more than 1 Group listed in the byline) and/or Subgroup (eg, Steering Committee)
Hailing	Dou			Zhengzhou Central Hospital		Data collection	
Kun	Wang			Zhengzhou First People's Hospital		Data collection	
Guangjun	Nie			Zibo Central Hospital		Data collection	
