# Supplementary material for: Efficacy and Safety of Ginkgo Diterpene Lactone Meglumine in Acute Ischemic Stroke: A Randomized Clinical Trial
Source: JAMA Netw Open. 2023 Aug 14;6(8):e2328828. doi: 10.1001/jamanetworkopen.2023.28828 (PMC10425831; doi:10.1001/jamanetworkopen.2023.28828)
Supplement: Supplement 4. — Data Sharing Statement [file jamanetwopen-e2328828-s004.pdf]

Data Sharing Statement

Zhang. Efficacy and Safety of Ginkgo Diterpene Lactone Meglumine in Acute Ischemic Stroke. *JAMA Netw Open*. Published August 14, 2023. doi:10.1001/jamanetworkopen.2023.28828

Data
Data available: No
